# Infiltration of meningeal macrophages into the Virchow–Robin space after ischemic stroke in rats: Correlation with activated PDGFR-β-positive adventitial fibroblasts

**DOI:** 10.3389/fnmol.2022.1033271

**Published:** 2022-12-30

**Authors:** Tae-Ryong Riew, Ji-Won Hwang, Xuyan Jin, Hong Lim Kim, Mun-Yong Lee

**Affiliations:** ^1^Department of Anatomy, Catholic Neuroscience Institute, College of Medicine, The Catholic University of Korea, Seoul, South Korea; ^2^Department of Biomedicine and Health Sciences, College of Medicine, The Catholic University of Korea, Seoul, South Korea; ^3^Integrative Research Support Center, Laboratory of Electron Microscope, College of Medicine, The Catholic University of Korea, Seoul, South Korea

**Keywords:** platelet-derived growth factor beta, Virchow–Robin space, perivascular fibroblasts, ischemic stroke, meningeal macrophages

## Abstract

Macrophages play a crucial role in wound healing and fibrosis progression after brain injury. However, a detailed analysis of their initial infiltration and interaction with fibroblasts is yet to be conducted. This study aimed to investigate the possible route for migration of meningeal macrophages into the ischemic brain and whether these macrophages closely interact with neighboring platelet-derived growth factor beta receptor (PDGFR-β)-positive adventitial fibroblasts during this process. A rat model of ischemic stroke induced by middle cerebral artery occlusion (MCAO) was developed. In sham-operated rats, CD206-positive meningeal macrophages were confined to the leptomeninges and the perivascular spaces, and they were not found in the cortical parenchyma. In MCAO rats, the number of CD206-positive meningeal macrophages increased both at the leptomeninges and along the vessels penetrating the cortex 1 day after reperfusion and increased progressively in the extravascular area of the cortical parenchyma by 3 days. Immunoelectron microscopy and correlative light and electron microscopy showed that in the ischemic brain, macrophages were frequently located in the Virchow–Robin space around the penetrating arterioles and ascending venules at the pial surface. This was identified by cells expressing PDGFR-β, a novel biomarker of leptomeningeal cells. Macrophages within penetrating vessels were localized in the perivascular space between smooth muscle cells and PDGFR-β-positive adventitial fibroblasts. In addition, these PDGFR-β-positive fibroblasts showed morphological and molecular characteristics similar to those of leptomeningeal cells: they had large euchromatic nuclei with prominent nucleoli and well-developed rough endoplasmic reticulum; expressed nestin, vimentin, and type I collagen; and were frequently surrounded by collagen fibrils, indicating active collagen synthesis. In conclusion, the perivascular Virchow–Robin space surrounding the penetrating vessels could be an entry route of meningeal macrophages from the subarachnoid space into the ischemic cortical parenchyma, implying that activated PDGFR-β-positive adventitial fibroblasts could be involved in this process.

## 1. Introduction

Fibroblast-like cells form a dense fibrotic scar in the lesion core after central nervous system (CNS) injury (Fernandez-Klett and Priller, 2014). In the CNS, fibroblast-like cells exist in the meninges and perivascular Virchow–Robin space between the vascular smooth muscle cells and glia limitans (Fernandez-Klett et al., 2013; Soderblom et al., 2013; Fernandez-Klett and Priller, 2014; Vanlandewijck et al., 2018; Xu and Yao, 2021). We have recently demonstrated that platelet-derived growth factor beta receptor (PDGFR)-β-positive fibroblasts form a cellular network throughout the leptomeninges. They are also observed in the leptomeningeal sheath of the subarachnoid vessels, which continue with the adventitia of larger caliber vessels in the cortical parenchyma. Importantly, this provides evidence that PDGFR-β can be used as a novel biomarker for leptomeningeal cells and perivascular fibroblasts in the brain parenchyma (Riew et al., 2018, 2020).

In addition to leptomeningeal fibroblasts, the leptomeninges harbor diverse immune cells including macrophages, dendritic cells, and lymphoid cells (Jordão et al., 2019; Kierdorf et al., 2019; Derk et al., 2021). Leptomeningeal macrophages, along with perivascular and choroid plexus macrophages, are key components of the brain-resident immune system and actively participate in various processes in normal and diseased states (Lapenna et al., 2018; Kierdorf et al., 2019; Yang et al., 2019; Derk et al., 2021). Further, they regulate the acute injury response by influencing the severity of neuroinflammation and fibrosis (Derk et al., 2021). Several studies have reported the presence of macrophages in the Virchow–Robin space surrounding the pial and penetrating arterioles into the cortical parenchyma (Koizumi et al., 2019; Yang et al., 2019). Particularly, experiments using the systemic injection of fluorescent monocytes after brain ischemia showed an entry route of monocytes into the ischemic tissue (i.e., from the leptomeninges along the penetrating vessels to the deeper cortical zone), suggesting that the vasculature is permissive to cell migration (Riew et al., 2018). However, the exact route for brain infiltration of leptomeningeal macrophages, especially along the path of the penetrating vessels into the ischemic cortex, remains to be determined.

Increasing evidence has shown the dynamic interactions between macrophages and fibroblasts in wound healing and fibrosis progression (Leibovich and Ross, 1975; Duffield et al., 2005; Nishida et al., 2008; Lucas et al., 2010; Nikolic-Paterson et al., 2014; Knipper et al., 2015; Zhu et al., 2015, 2017). Our previous study also showed that activated PDGFR-β-fibroblasts frequently interact closely with brain macrophages after brain injury (Riew et al., 2018). This study aimed to investigate the possible route for migration of meningeal macrophages into the ischemic brain and whether they closely interact with neighboring PDGFR-β-positive adventitial fibroblasts in this process. We hypothesized that leptomeningeal macrophages migrate into the ischemic cortex along the perivascular space of the penetrating vessels, in which they closely interact with perivascular fibroblasts that activate and transform into the morphology of leptomeningeal fibroblasts.

Toward this goal, the localization of macrophages expressing CD206, a specific marker of perivascular macrophages and meningeal macrophages (Mrdjen et al., 2018; Van Hove et al., 2019), was examined. Particularly, we investigated their possible interactions with neighboring PDGFR-β-positive fibroblasts in the leptomeninges and vessels penetrating the cortical parenchyma in the acute phase of stroke induced by middle cerebral artery occlusion (MCAO) in rats. In addition, the changes in the ultrastructural and molecular features of PDGFR-β-positive perivascular fibroblasts in the ischemic cortex were analyzed. Considering that both macrophages and perivascular fibroblasts reside in close proximity within the perivascular space, an ultrastructural study using the immunoperoxidase method combined with correlative light and electron microscopy approach was performed to identify the exact relationship between these cells.

## 2. Materials and methods

### 2.1. Animal preparation

Adult male Sprague–Dawley rats (250–300 g, OrientBio, Seongnam, Republic of Korea) were used. The animals were housed in groups of three per cage in a controlled environment at a constant temperature (22 ± 5°C) and humidity (50 ± 10%) with food (gamma ray-sterilized diet) and water (autoclaved tap water) available *ad libitum*. They were maintained on a 12-h light/dark cycle. Transient focal cerebral ischemia was induced using the intraluminal thread method as described previously (Longa et al., 1989; Shin et al., 2011). Briefly, a 3–0 rounded-tip nylon suture was inserted into the right common carotid artery and advanced through the internal carotid artery until it occluded the MCA. After 45 min of occlusion, cerebral blood flow was reinstated by withdrawal of the nylon suture from the MCA. Body temperatures (measured rectally) were maintained at 37.5 ± 0.3°C with a heating pad during and after the surgical procedure. Sham-operated rats underwent the same experimental process, except for MCAO using nylon sutures. The animals in the ischemic and sham-operated groups (*n* = 12 rats/group) were allowed to recover for 1 and 3 days after reperfusion, respectively. They were then perfused transcardially with 4% paraformaldehyde in 0.1 M phosphate buffer (PB; pH 7.4) after anesthetized with zolazepam (20 mg/kg i.p.) and xylazine (7.5 mg/kg i.p.). The brain tissues were equilibrated with 30% sucrose in 0.1 M PB and frozen until use.

All procedures and provisions for animal care were in accordance with the Laboratory Animals Welfare Act, the Guide for the Care and Use of Laboratory Animals, and the Guidelines and Policies for Rodent Survival Surgery provided by the IACUC (Institutional Animal Care and Use Committee) at the College of Medicine of The Catholic University of Korea (Approval number: CUMS-2020-0041-01). The IACUC and the Department of Laboratory Animals in the Catholic University of Korea, Songeui Campus, accredited the Korea Excellence Animal Laboratory Facility from the Korea Food and Drug Administration in 2017 and acquired full Association for Assessment and Accreditation of Laboratory Animal Care International accreditation in 2018. All efforts were made to minimize animal suffering and to reduce the number of animals used.

### 2.2. Immunohistochemistry

For double or triple immunofluorescence immunohistochemistry, 25 μm-thick free-floating sections were blocked in blocking buffer (a mixture of 10% normal serum, 1% bovine serum albumin, and 0.1% triton or a mixture of 3% normal serum, 1% bovine serum albumin, and 0.5% triton) and then incubated at 4°C overnight with a mix of primary antibodies. The primary antibodies used are summarized in Table 1. Antibody staining was visualized after a 2-h incubation period with the following secondary antibodies: Cy3-conjugated donkey anti-goat/chicken/mouse antibody (1:2000; Jackson ImmunoResearch, Westgrove, PA, United States), Alexa Fluor 488-tagged donkey anti-rabbit antibody (1:300; Thermo Fisher, Waltham, MA, United States), and Alexa Fluor 647-tagged donkey anti-mouse/goat/chicken antibody (1:300; Thermo Fisher). Negative staining controls for the triple immunofluorescence were performed by omission of the primary or secondary antibodies. In addition, the results of triple labeling were compared with those of single and double labeling of all combinations of antibodies to ensure a clear interpretation of the results. Cell nuclei were counterstained with DAPI (4′,6-diamidino-2′-phenylindole, 1:2000; Roche, Mannheim, Germany) for 10 min. Slides were viewed under a confocal microscope (LSM 900 with Airyscan; Carl Zeiss Co. Ltd., Oberkochen, Germany) equipped with four lasers (Diode 405, Argon 488, HeNe 543, and HeNe 633) under constant viewing conditions. The size of images ranged from 512 × 512 pixels to 1,024 × 1,024 pixels. Images were converted to the TIFF format, and contrast levels and sizes were adjusted using Adobe Photoshop. Three-dimensional reconstruction and orthogonal view of confocal images were achieved using Zen 3.0 blue (Carl Zeiss Co. Ltd.) and IMARIS (Bitplane, Zurich, Switzerland).

**Table 1:** Details of primary antibodies used.

Antigen	Marker	Dilution	Manufacturing details	Host
CD206	Border-associated macrophage	1:300	R&D systems, Minneapolis, MN, United States, AF2535	Goat
Collagen I	Extracellular matrix (fibrotic scar)	1:100	Abcam, Cambridge, United Kingdom, ab34710	Rabbit
Collagen IV	Extracellular matrix (fibrotic scar)	1:100	Bio-Rad, Hercules, CA, United States, 134001	Goat
Glial fibrillary acidic protein (GFAP)	Astrocyte	1:500	Merck, Darmstadt, Germany, AB5541	Chicken
Ionized calcium-binding adaptor molecule 1 (Iba1)	Microglia, macrophage	1:500	Wako Pure Chemical Co., Osaka, Japan, 019–19741	Rabbit
		1:400	Abcam, Cambridge, United Kingdom, ab14917	Goat
Lymphatic vessel endothelial hyaluronan receptor 1 (Lyve1)	Border-associated macrophage	1:100	Abcam, Cambridge, United Kingdom, ab5076	Rabbit
Nestin	Type VI intermediate filament, activated fibroblast	1:500	Bio-Rad, Hercules, CA, United States, 6625–1010	Mouse
Platelet-derived growth factor receptor β (PDGFR-β)	Meningeal/perivascular fibroblast	1:200	Abcam, Cambridge, United Kingdom, ab32570	Rabbit
		1:100	R&D systems, Minneapolis, MN, United States, AF1042	Goat
Rat endothelial cell antigen-1 (RECA-1)	Endothelium (blood vessels)	1:200	Bio-Rad, Hercules, CA, United States, MCA970GA	Mouse
Vimentin	Type III intermediate filament, activated fibroblast	1:500	Merck, Darmstadt, Germany, AB5733	Chicken

### 2.3. Quantitative image analysis and statistics

**Figure 1:**
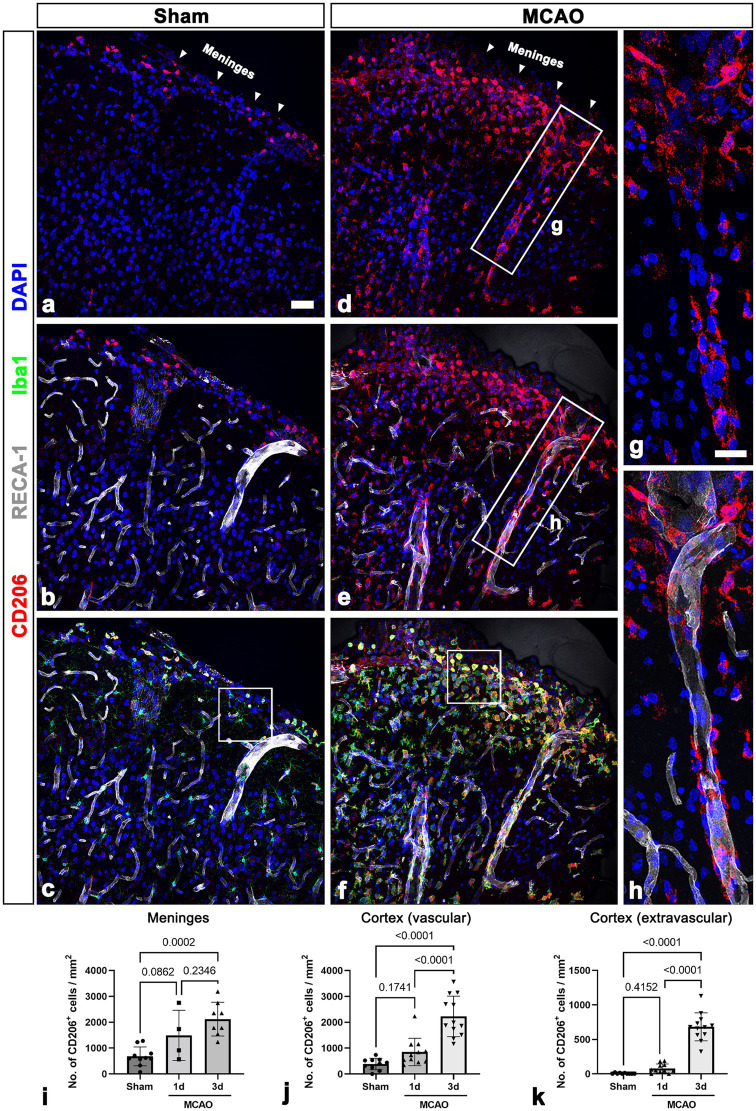
CD206-positive macrophages in the leptomeninges and the underlying cerebral cortex in sham-operated and MCAO rats. **(A–C)** Triple labeling for CD206, the endothelial cell marker RECA-1, and Iba1 in control sections showing that CD206/Iba1 double-labeled macrophages are occasionally observed in the leptomeninges (arrowheads in a) and large cortical penetrating vessels but not in the cortical parenchyma. **(D–F)** Triple labeling for CD206, RECA-1, and Iba1 in the cortex reperfused for 3 days showing that CD206/Iba1 double-labeled macrophages have accumulated at the leptomeninges (arrowheads in **D**) and in the superficial cortical parenchyma. Note that these macrophages are associated with penetrating large-sized cortical vessels. **(G,H)** Higher magnification views of the boxed areas in d and e, respectively. CD206-positive macrophages with elongated morphologies are located along the wall of penetrating cortical vessels. **(I–K)** Quantitative temporal analysis showing that the number of CD206-positive macrophages is significantly increased in the leptomeninges and cortex including the vascular and surrounding extravascular areas 3 days after ischemia (*n* = 3 sections from 4 to 12 rats per time points, one-way ANOVA with Tukey’s multiple comparison test). Data are expressed as the mean ± SEM, and numbers on each bar graph indicate *p* values. Scale bar = 50 μm for a–f, 25 μm for **(G,H)**.

For quantitative analysis, confocal images were obtained with a 40× objective lens with constant viewing settings for each analysis. To compare the number of CD206(+) and Lyve1(+) cells in different CNS compartments, 319.45 × 319.45-μm (Figure 1) or 607.08 × 607.08-μm (Supplementary Figure 1) images of 13-μm Z-stacks with 0.5-μm optical sections were used. Cells with intact DAPI staining were counted, and the numbers were normalized to the area (μm^2^) of each compartment. To compare the distribution of PDGFR-β and CD206 occupying the vascular area, merged images of 319.45 × 319.45 μm and 11-μm Z-stacks with 0.5-μm optical sections were used, and the lengths of rat endothelial cell antigen-1 (RECA-1)(+) vascular structures in the cortical parenchyma were measured. The mean fluorescence intensity profiles of PDGFR-β, collagen IV, nestin, and vimentin were obtained using Zen 3.0 blue edition. The number of animals used for each analysis is indicated in the figure legends. Data were analyzed using Student’s t-test or one-way analysis of variance followed by Tukey’s multiple comparison test. All statistical analyses were performed using Prism 7 (GraphPad Software Inc., San Diego, CA, United States). *p* values <0.05 indicated significance. The exact *p* values are indicated in each figure.

### 2.4. Immunoelectron microscopy

Five sham-operated rats and five experimental rats were euthanized 3 days after MCAO for use of their brains in immunoelectron microscopy or the correlative light and electron microscopy study, respectively. For pre-embedding immunoelectron microscopy, floating vibratome sections (50 μm thick) from rat brains were blocked with 10% normal goat serum and 1% bovine serum albumin in 0.01 M PBS for 1 h. The sections were then immunostained with rabbit anti-PDGFR-β (1:200; Abcam) overnight at 4°C. Thereafter, the sections were incubated with peroxidase-conjugated goat anti-rabbit IgG (1:100; Jackson ImmunoResearch) for 1.5 h and then visualized using 0.05% 3,3′-diaminobenzidine tetrahydrochloride as a chromogen. After fixating, dehydrating, and embedding in Epon 812 (Polysciences, Warrington, PA, United States), areas of interest were excised and glued onto resin blocks. Ultrathin sections (70-nm thick) were obtained and observed under an electron microscope (JEM 1010, JEOL, Tokyo, Japan) with uranyl acetate staining.

For the correlative light and electron microscopy study, the skull and dura were removed from the arachnoid using fine forceps, and square incisions were made on the surface of the cerebral hemispheres. Small blocks of tissue 1–2 mm in depth including the leptomeninges and cerebral cortex were obtained and cryoprotected with 2.3 M sucrose in 0.1 M PB and frozen in liquid nitrogen. Semi-thin cryosections (3-μm thick) were cut at −100°C with a glass knife in a Leica EM UC7 ultramicrotome equipped with an FC7 cryochamber (Leica, Wetzlar, Germany). The sections were triple labeled at 4°C overnight using a mix of mouse anti-RECA1 (1:200, Bio-Rad), rabbit anti-PDGFR-β (1:200; Abcam), and goat anti-CD206 (1:300, R&D systems) antibodies.

Antibody staining was visualized using Cy3-conjugated donkey anti-goat (1:2000, Jackson ImmunoResearch), Alexa 488-tagged donkey anti-rabbit (1:300; Thermo Fisher), and Alexa Fluor 647-tagged donkey anti-mouse (1:500, Abcam) antibodies. Sections were counterstained with DAPI for 10 min. Coverslipped sections were examined with a confocal microscope and photographed at ×200, ×400, and × 630 magnifications with a differential interference contrast setting to identify specific areas for succeeding electron microscopy. After the sections were removed from the coverslips, the tissues were prepared for electron microscopy as described previously (Kim et al., 2021; Riew et al., 2021).

## 3. Results

### 3.1. CD206 as a biomarker of leptomeningeal macrophages

We first validated two well-known markers for CNS border-associated macrophages (BAMs): CD206 and lymphatic vessel endothelial hyaluronan receptor 1 (Lyve1; Jordão et al., 2019; Karam et al., 2022; Masuda et al., 2022). Double labeling with either CD206 or Lyve1 and ionized calcium-binding adaptor molecule 1 (Iba1) showed that both markers were present in Iba1-positive macrophage-like phenotype with amoeboid morphology, but they exhibited different degrees of coexistence with Iba1. CD206 and Iba1 generally overlapped within leptomeninges and cortical vessels, while most Lyve1-positive cells corresponded to only a subpopulation of Iba1-positive cells (Supplementary Figures 1A–F). This observation was confirmed by results of quantitative analysis that the proportion of CD206/Iba1 double-labeled macrophages was higher than that of Lyve1/Iba1 double-labeled macrophages (Supplementary Figures 1J, K). Moreover, double labeling with CD206 and Lyve1 revealed that Lyve1 expression was restricted to a subset of CD206-positive macrophages within leptomeninges (Supplementary Figures 1G–I). In addition, analysis of Lyve1 distribution in different brain regions supported our findings that Lyve1(+) cells are a subpopulation of CD206(+) cells (Karam et al., 2022). These data indicated that CD206 may be a more suitable marker for detecting leptomeningeal macrophages than Lyve1.

### 3.2. Spatial localization of CD206-positive macrophages within the infarct area in the acute phase of cerebral ischemia

Localization of leptomeningeal macrophages was assessed in sham-operated rats and 3 days after ischemia in MCAO rats using triple immunofluorescence with CD206; Iba1; and RECA-1, a vascular endothelial cell marker. In sham-operated rats, CD206/Iba1 double-labeled macrophages were occasionally observed in the leptomeninges and large cortical penetrating vessels, but they were not detected in the cortical parenchyma (Figures 1A–C; Supplementary Figures 2A–D). In MCAO rats, the number of CD206/Iba1 double-labeled macrophages was increased both at the leptomeninges and within the ischemic cortex 3 days after reperfusion. Further, these macrophages were associated with penetrating large cortical vessels (Figures 1D–F). Higher magnification images revealed that CD206-positive macrophages with elongated morphologies were located along the wall of penetrating cortical vessels (Figures 1G, H). This suggested that leptomeningeal macrophages migrate along the penetrating vessels into the ischemic cortex. In addition, these macrophages appeared in the cortical parenchyma and were particularly accumulated in the superficial cortical parenchyma in close proximity to the pia mater (Figures 1D–F, Supplementary Figures 2E–H). This observation was confirmed in the results of quantification analysis that the number of CD206-positive macrophages was increased in both the leptomeninges and the ischemic cortex over a 3-day period after ischemia (Figures 1I–K). In the cortical parenchyma, the number of both macrophages attached to the vasculature and those free in the cortical parenchyma was significantly increased 3 days after ischemia (Figures 1J, K).

### 3.3. Temporal expression profiles and molecular characterization of PDGFR-β in the leptomeninges and the underlying cerebral cortex in the acute phase of cerebral ischemia

**Figure 2:**
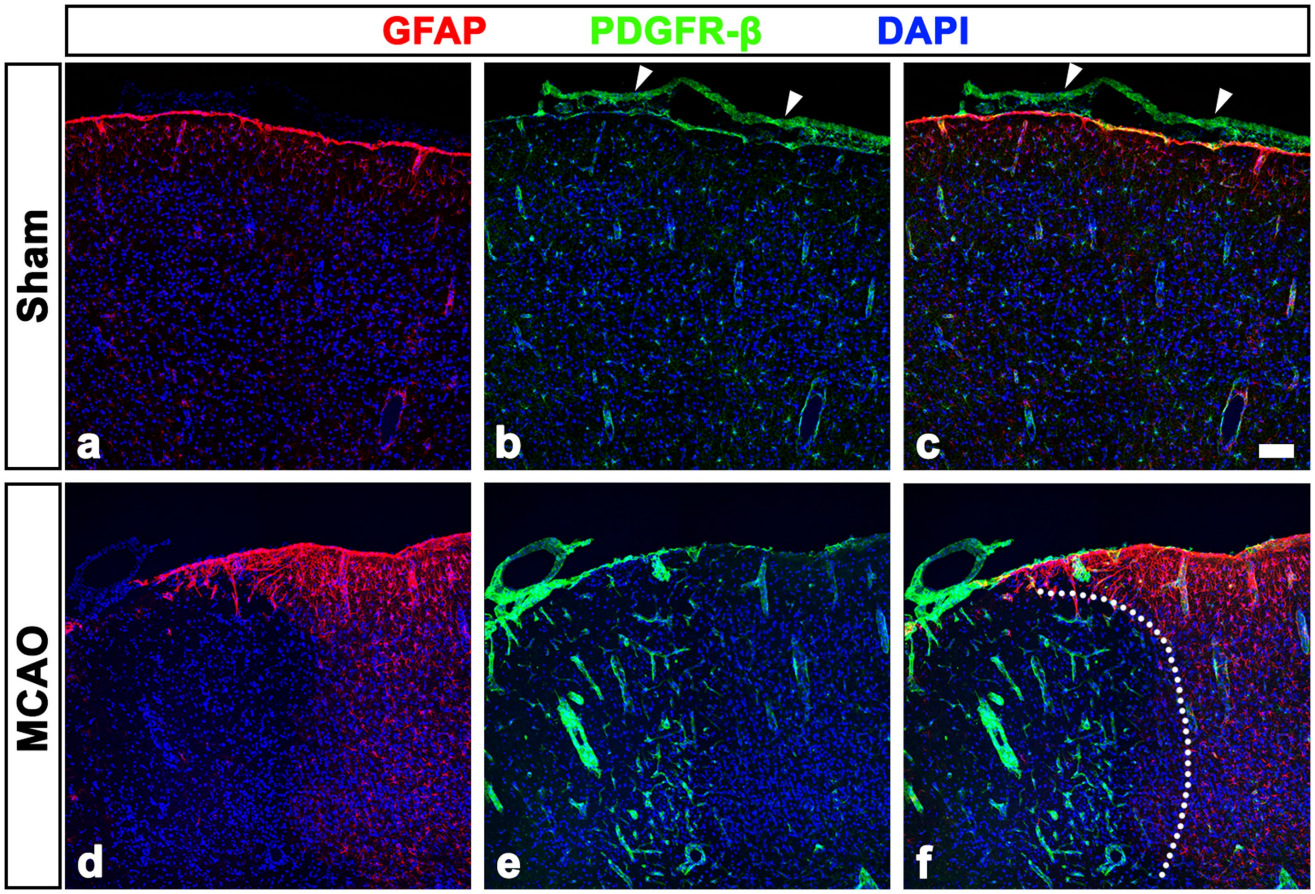
PDGFR-β expression profiles in the leptomeninges and underlying cortex of sham-operated and MCAO rats. **(A–C)** Double labeling for PDGFR-β and GFAP in control sections showing that PDGFR-β is expressed in the leptomeninges (arrowheads in **B** and **C**) and cortical vessels but not in the GFAP-positive glia limitans superficialis covering the cortical surface. **(D–F)** Double labeling for PDGFR-β and GFAP in the cortex reperfused for 3 days showing that PDGFR-β expression is prominent in vascular profiles in the lesion core, where GFAP immunoreactivity is absent. The broken line indicates the border between the lesion core and the peri-lesional area. Scale bar = 100 μm for **(A–F)**.

**Figure 3:**
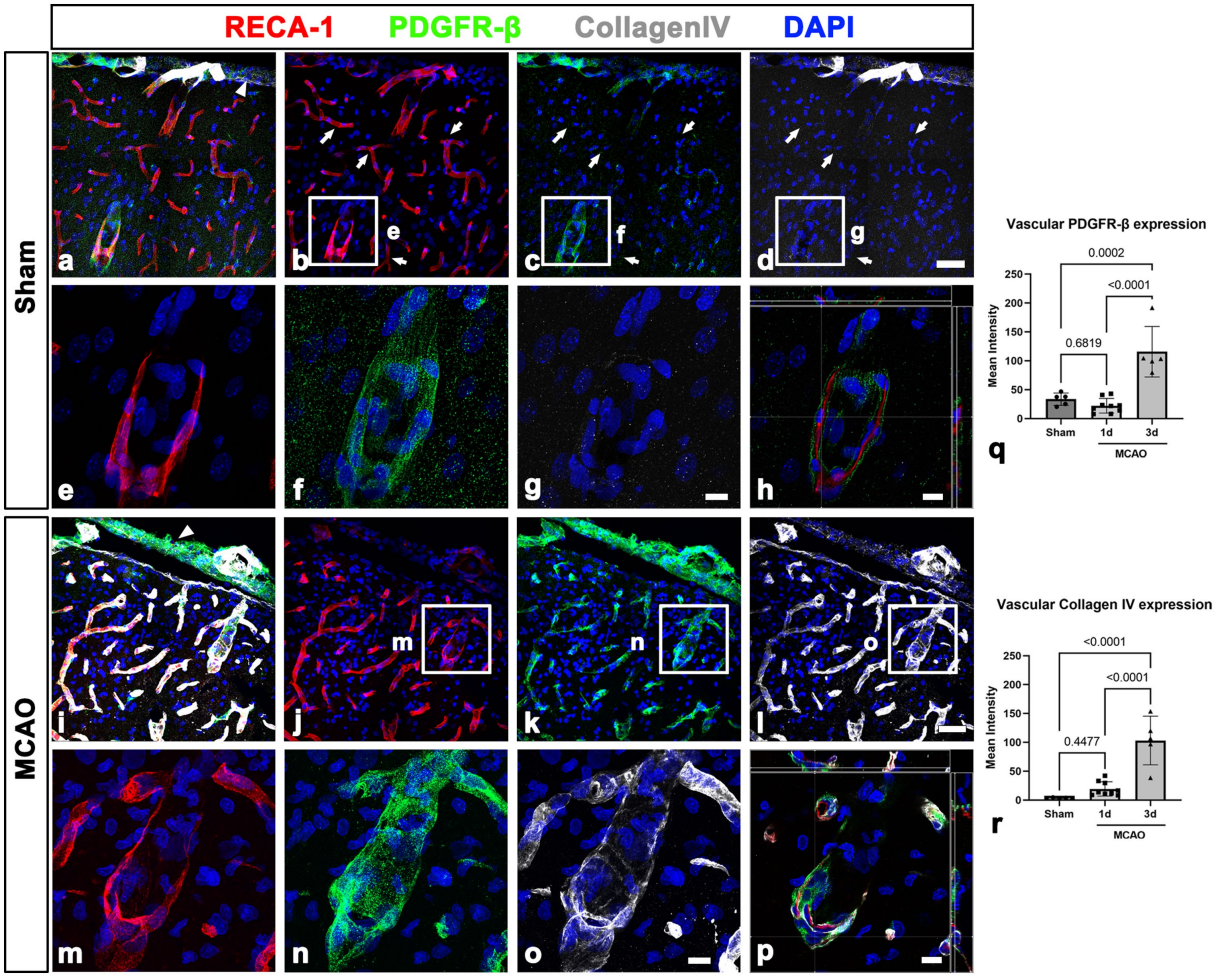
PDGFR-β expression profiles and their spatial relationship with collagen in the leptomeninges and underlying cortex in sham-operated and MCAO rats. **(A–H)** Triple labeling for PDGFR-β, RECA-1, and collagen IV. **(A–D)** In sham-operated rats, collagen IV is detected in PDGFR-β-positive leptomeninges (arrowhead in **A**) including in their vessels but not in PDGFR-β-positive larger cortical vessels. Note the absence of immunoreactivities for PDGFR-β and collagen in the capillary-like vessels (arrows in **B–D**). **(E–G)** Higher magnification views of the boxed areas in **(B–D)**, respectively. **(H)** The orthogonal view of boxed areas in **(B–D)**. **(I–L)** At 3 days after MCAO, PDGFR-β and collagen IV have overlapping distributions within both leptomeninges (arrowhead in **E**) and most cortical blood vessels. **(M–O)** Higher magnification views of the boxed areas in **(J–L)**, respectively. **(P)** The orthogonal view of boxed areas in **(J–L)**. **(Q,R)** Quantitative analysis showing that the relative intensities of both PDGFR-β and collagen IV in cortical blood vessels at day 3 after reperfusion are significantly higher in MCAO rats than in sham-operated rats (*n* = 5–7 sections from 6 to 8 rats per time points, one-way ANOVA with Tukey’s multiple comparison test). Data are expressed as the mean ± SEM, and numbers on each bar graph indicate *p* values. Scale bars = 50 μm for **(A–D,I–L)** 10 μm for **(E–H,M–P)**.

We next examined PDGFR-β-positive cells in the sham-operated and MCAO rats 3 days after reperfusion, using double labeling for PDGFR-β and GFAP. In sham-operated rats, PDGFR-β was expressed in the leptomeninges and cortical vessels but not in the GFAP-positive glia limitans superficialis covering the cortical surface (Figures 2A–C), consistent with the findings of our previous study (Riew et al., 2020). Three days post-reperfusion, PDGFR-β expression was prominent in vascular profiles in the lesion core, which was clearly demarcated by the absence of GFAP immunoreactivity (Figures 2D–F), as described previously (Mu et al., 2016; Jin et al., 2018; Riew et al., 2018). PDGFR-β expression was further characterized in the leptomeninges and underlying cortical parenchyma by triple labeling for PDGFR-β, RECA-1, and collagen IV. In sham-operated rats, PDGFR-β was expressed in both the leptomeninges including their vessels and the larger cortical parenchymal vessels, while collagen was detected in leptomeninges, but its amount was negligible in cortical vessels (Figures 3A–H), as reported previously (Riew et al., 2020). The capillary-like vessels in the cortical parenchyma had no significant immunoreactivities for PDGFR-β and collagen (Figures 3A–D). Three days post-reperfusion, the distribution of PDGFR-β and collagen IV was overlapping within most cortical blood vessels and within the leptomeninges (Figures 3I–P), consistent with previous studies showing the increased expression of vascular collagen IV after ischemic stroke (Ji and Tsirka, 2012; Hawkes et al., 2013; Michalski et al., 2020).

**Figure 4:**
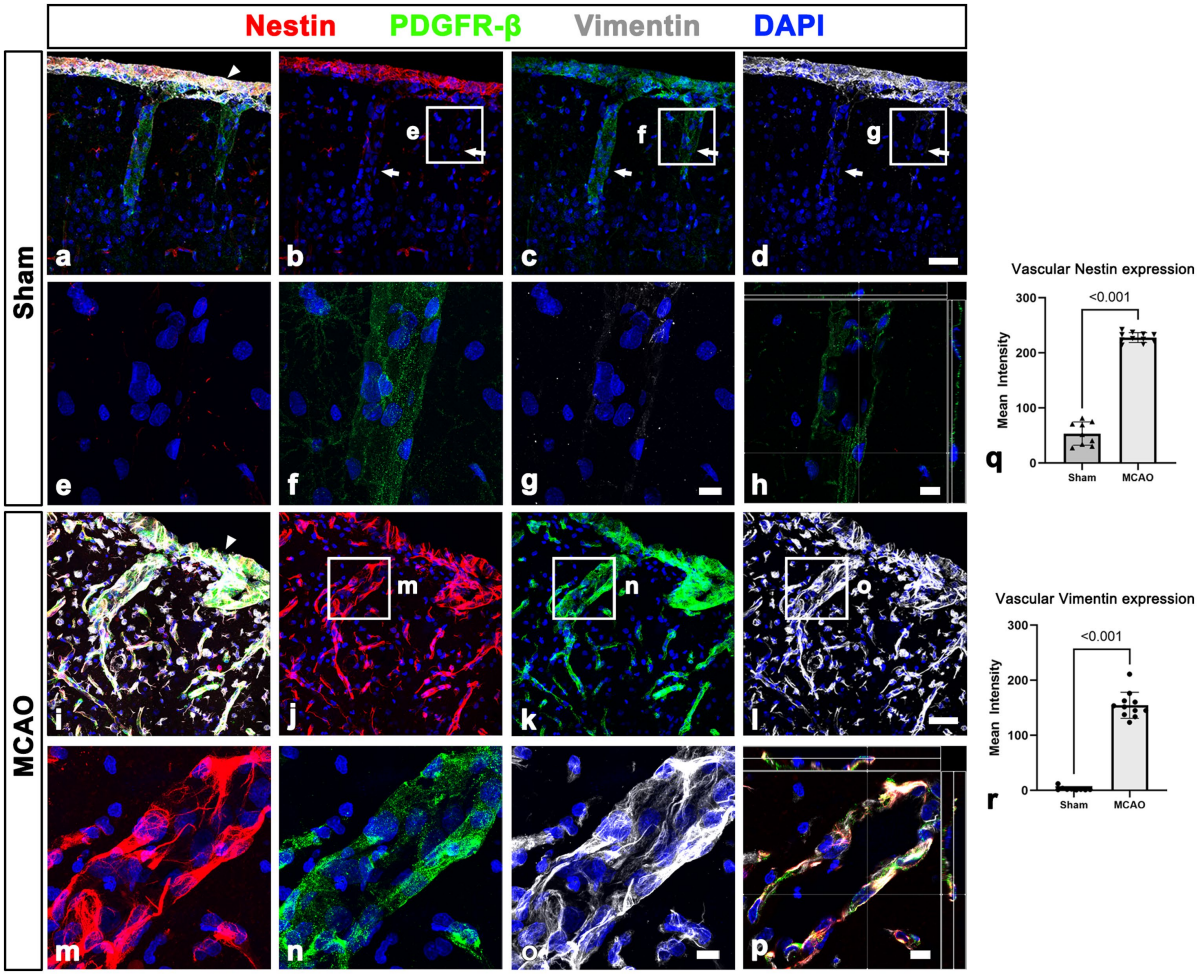
Molecular characterization of PDGFR-β expression in the leptomeninges and underlying cortex in sham-operated and MCAO rats. **(A–H)** Triple labeling for PDGFR-β, nestin, and vimentin. **(A–D)** In sham-operated rats, the two intermediate filament proteins nestin and vimentin are observed in the leptomeninges (arrow in **A**) but not in larger cortical vessels (arrows in **B–D**), despite both being immunoreactive for PDGFR-β. **(E–G)** Higher magnification views of the boxed areas in **(B–D)**, respectively. **(H)** The orthogonal view of boxed areas in **(B–D)**. **(I–L)** At 3 days after MCAO, PDGFR-β and collagen IV had overlapping distributions within both leptomeninges (arrowhead in **E**) and most cortical blood vessels. **(M–O)** Higher magnification views of the boxed areas in **(J–L)**, respectively. **(P)** The orthogonal view of boxed areas in **(J–L)**. **(Q,R)** Quantitative analysis showing that the relative intensities of nestin and vimentin in cortical blood vessels are significantly higher 3 days after reperfusion in MCAO rats than in control rats (*n* = 5–8 sections from 5 to 11 rats per time points, Student’s t-test). Data are expressed as the mean ± SEM, and numbers on each bar graph indicate p values. Scale bars = 50 μm for **(A–D,I–L)** 10 μm for **(E–H,M–P)**.

This finding was further supported by results of quantitative analysis that the relative intensities of both PDGFR-β and collagen IV in cortical blood vessels in the lesion core at day 3 were significantly higher in MCAO rats than in sham-operated rats (Figures 3Q, R). The molecular characteristics of PDGFR-β-positive cortical vessels in the ischemic cortical parenchyma was next examined *via* triple labeling for PDGFR-β and the two intermediate filament proteins nestin and vimentin. In sham-operated rats, leptomeningeal PDGFR-β-positive cells expressed nestin and vimentin, while PDGFR-β-positive cortical vessels did not express these proteins (Figures 4A–H), as reported previously (Riew et al., 2020). In MCAO rats, PDGFR-β, nestin, and vimentin showed overlapping expression in the cortical blood vessels 3 days after reperfusion (Figures 4I–P). A quantitative comparison of the relative intensities of both nestin and vimentin in cortical blood vessels revealed that they were significantly higher in the lesion core at day 3 in MCAO rats than in sham-operated rats (Figures 4Q, R). Further, we performed double labeling for PDGFR-β and type I collagen, which are commonly used as molecular markers of perivascular fibroblasts as their expression is enriched during the production of collagen I (Kelly et al., 2016; Bonney et al., 2022; Dorrier et al., 2022). As indicated in Supplementary Figures 3A–F, the distribution of PDGFR-β and collagen I was overlapping within the leptomeninges and cortical blood vessels in both sham-operated and MCAO rats.

### 3.4. Spatial relationship between leptomeningeal macrophages and PDGFR-β-positive cells within the blood vessels penetrating the cortical parenchyma in the acute phase of cerebral ischemia

**Figure 5:**
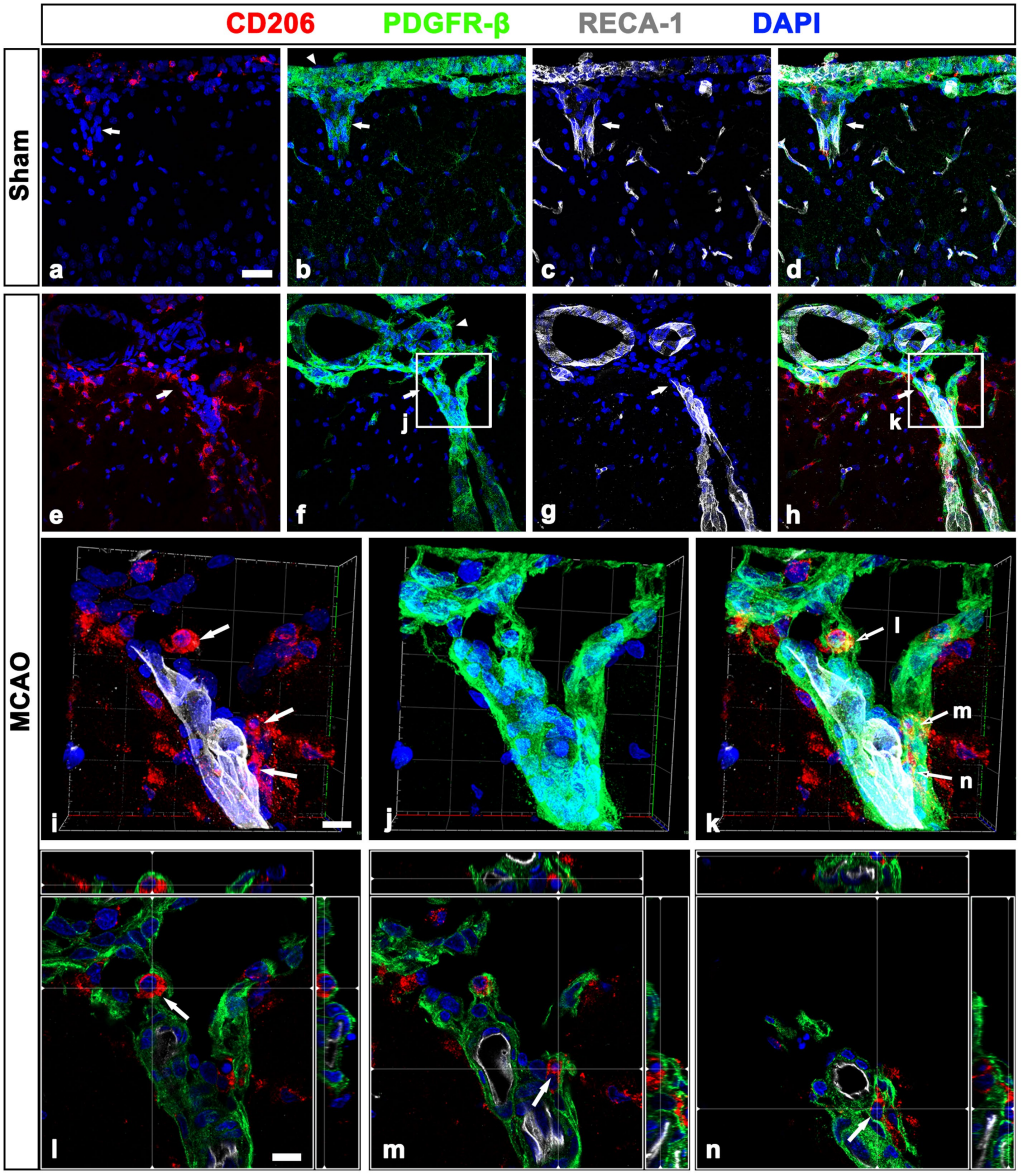
Relationship between leptomeningeal macrophages and PDGFR-β(+) cells within penetrating vessels in sham-operated and MCAO rats. **(A–H)**. Triple labeling for PDGFR-β, CD206, and RECA-1 in sham-operated **(A–D)** and MCAO rats at day 3 after reperfusion **(E–H)**. CD206-labeled macrophages are rarely observed in PDGFR-β-positive penetrating cortical vessels of sham-operated rats (arrows in **A–D**), while several meningeal macrophages are noted within penetrating vessels (arrows in **E–H**) in MCAO rats. Arrowheads in **(B,F)** indicate the leptomeninges. **(I–K)** The three-dimensional magnified images of boxed areas in **(F,H)** (penetrating vessels close to the cortical surface) showing that meningeal macrophages (arrows in **I** and **K**) within penetrating vessels are localized in close proximity to PDGFR-β-positive cells. **(L–N)** Orthogonal views of three meningeal macrophages indicated by arrows in **(I,K)**, respectively, showing that they are located on the adluminal side of PDGFR-β-positive cells within penetrating vessels. Scale bar = 40 μm for **(A–H)**; 10 μm for **(I–N)**.

As shown in Figure 1H, CD206-positive meningeal macrophages were located along the wall of penetrating cortical vessels in MCAO rats. Thus, we determined the spatial relationship among meningeal macrophages, PDGFR-β-positive cells, and vascular endothelial cells within the penetrating cortical vessels. In sham-operated rats, CD206-labeled macrophages were rarely observed in penetrating large-sized cortical vessels, where they were closely associated with PDGFR-β-positive cells (Figures 5A–D). In cortical parenchyma reperfused for 3 days, several meningeal macrophages were noted within penetrating cortical vessels (Figures 5E–H). Higher magnification images using enhanced three-dimensional visualization showed that meningeal macrophages within penetrating vessels close to the cortical surface appeared to be localized in close proximity to PDGFR-β-positive cells, preferentially on the adluminal side of PDGFR-β-positive cells. This indicated that meningeal macrophages were located between endothelial cells and PDGFR-β-positive cells (Figures 5I–K). Orthogonal images further supported that meningeal macrophages were located on the adluminal side of PDGFR-β-positive cells within penetrating vessels, with these cells being closely associated with each other (Figures 5L–N). Despite the overlapping distribution of CD206-positive macrophages and PDGFR-β-expression within cortical penetrating vessels, these macrophages were located only in a small fraction of the vasculature occupied by PDGFR-β in both sham-operated and MCAO rats (Supplementary Figures 4A–H). This supported the possibility that vascular PDGFR-β expression may have been initiated prior to migration of meningeal macrophages. This finding was supported by the results of the semiquantitative analysis that among all PDGFR-β-positive vessels within the cortical penetrating vessels, the relative proportion of vascular profiles occupied by CD206 was 12.76% in sham and 40.75% in MCAO (Supplementary Figure 4I).

### 3.5. Ultrastructural relationship between leptomeningeal macrophages and PDGFR-β-positive cells within the leptomeninges and penetrating vessels of sham-operated and MCAO rats

**Figure 6:**
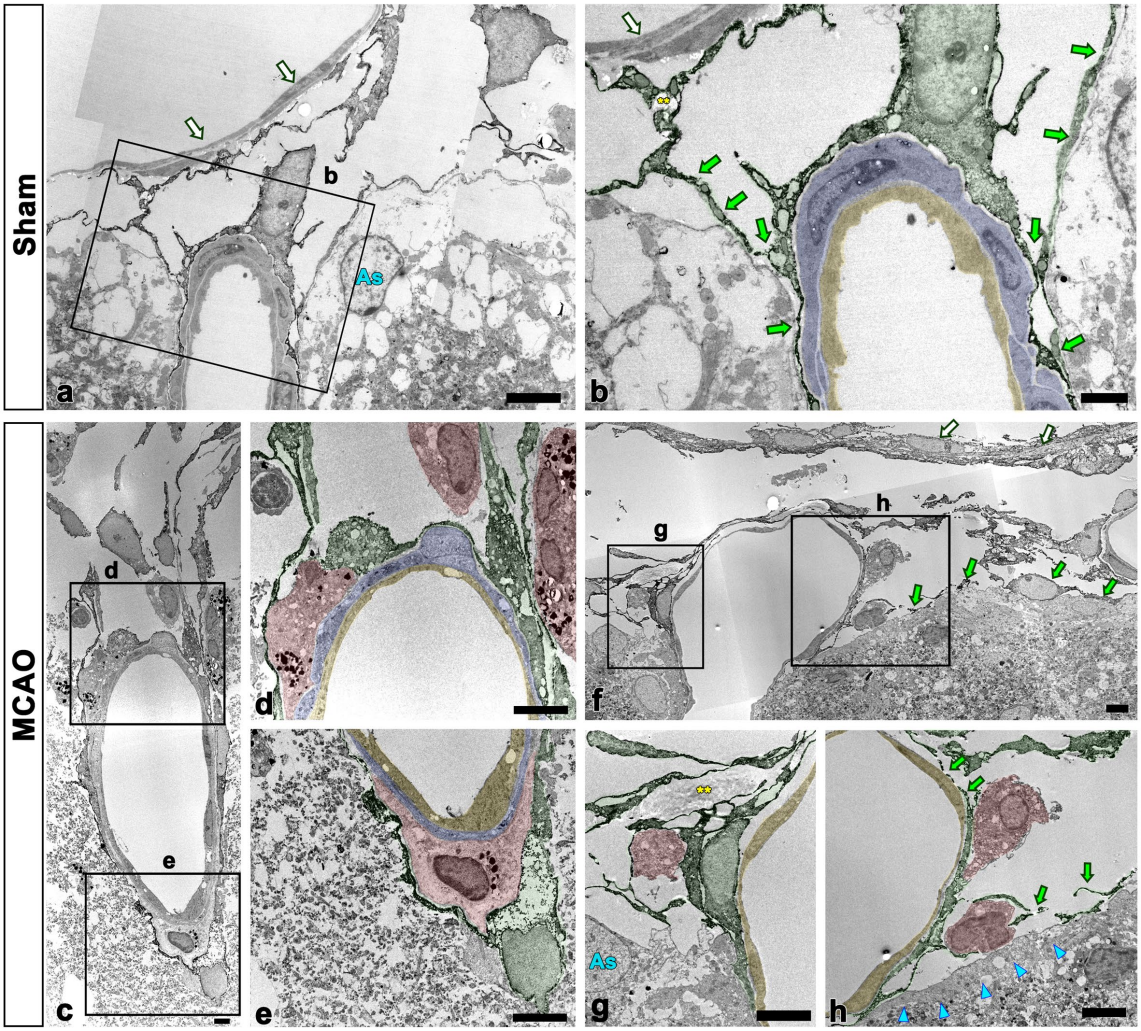
Ultrastructural relationship between macrophages and PDGFR-β(+) cells within the leptomeninges and penetrating cortical vessels. **(A,B)** Lower **(A)** and higher **(B)** magnification views in sham-operated rats showing that PDGFR-β-positive cells (shaded in green) ensheathing the pial vessels merge with the PDGFR-β-positive long slender processes (green arrows in **B**) of pial cells abutting the astrocytes (As in **A**) of the glia limitans superficialis. Note the location of PDGFR-β-positive processes on the abluminal side of smooth muscle cells (shaded in blue) and endothelial cells (shaded in yellow) of penetrating cortical vessels. Additionally, there is a lack of meningeal macrophages in the region where the penetrating vessel crossed the pia mater and entered the cortical parenchyma. **(C–H)** Close relationship between macrophages and PDGFR-β(+) cells in penetrating arterioles **(C–E)** and ascending venules **(F–H)**. The boxed areas in **(C)** are enlarged in **(D,E)**, and the boxed areas in **(F)** are enlarged in **(G,H)**. Note the meningeal macrophages (shaded in red) frequently appearing around the pial vessels on the cortical surface where the penetrating arterioles are branched or the ascending venules are converged. In addition, they are closely abutted to PDGFR-β-positive cell bodies and processes (shaded in green) located on the abluminal side of smooth muscle cells (shaded in blue) and endothelial cells (shaded in yellow) of both penetrating cortical vessels and pial vessels. Green arrows in **(F,H)** indicate PDGFR-β(+) pial cells abutting the astrocytes (As in **G**) of the glia limitans superficialis (cyan arrowheads in **H**). White arrows in **(A,B,F)** indicate PDGFR-β-negative cells composing the arachnoid mater. Yellow double asterisks in **(B,G)** indicate the collagen fibrils associated with PDGFR-β-positive cells. Scale bars = 5 μm for **(A,C–E,F–H)**; 2 μm for **(B)**.

To further clarify the spatial relationship between leptomeningeal macrophages and PDGFR-β-positive cells within vessels penetrating the cortical parenchyma, pre-embedding immunoelectron microscopy was performed. In sham-operated rats, PDGFR-β-positive cells ensheathing the leptomeningeal vessels merged with the PDGFR-β-positive long slender processes of pial cells that closely abutted the glia limitans superficialis delineating the cortical surface (Figures 6A, B), as previously described (Riew et al., 2020). In addition, PDGFR-β-positive processes were located on the abluminal side of smooth muscle cells of penetrating cortical vessels, indicating that they fit into the typical morphological features of perivascular fibroblasts (Riew et al., 2018, 2020). Meningeal macrophages were rarely detected in the region where penetrating vessels crossed the pia mater and entered the cortical parenchyma. In the cortex reperfused for 3 days, meningeal macrophages with amoeboid morphology were frequently observed in the leptomeninges and along the paths of the arterioles penetrating the infarcted cortex (Figures 6C–E). Particularly, they were commonly present where the penetrating arterioles entered the brain parenchyma at the pial surface (Figures 6C, D). Within penetrating arterioles, they were localized along the outer part of smooth muscle cells and were almost surrounded by PDGFR-β-positive cell bodies and processes, despite no membranous specialization being found between them (Figures 6C–E). In addition, macrophages were frequently found to be located in the region where the ascending venules converged into pial veins, which showed a funnel-shaped structure formed by the merging of the pial sheath ensheathing the pial veins and the pia mater abutting the glia limitans superficialis, both of which expressed PDGFR-β (Figures 6F–H).

**Figure 7:**
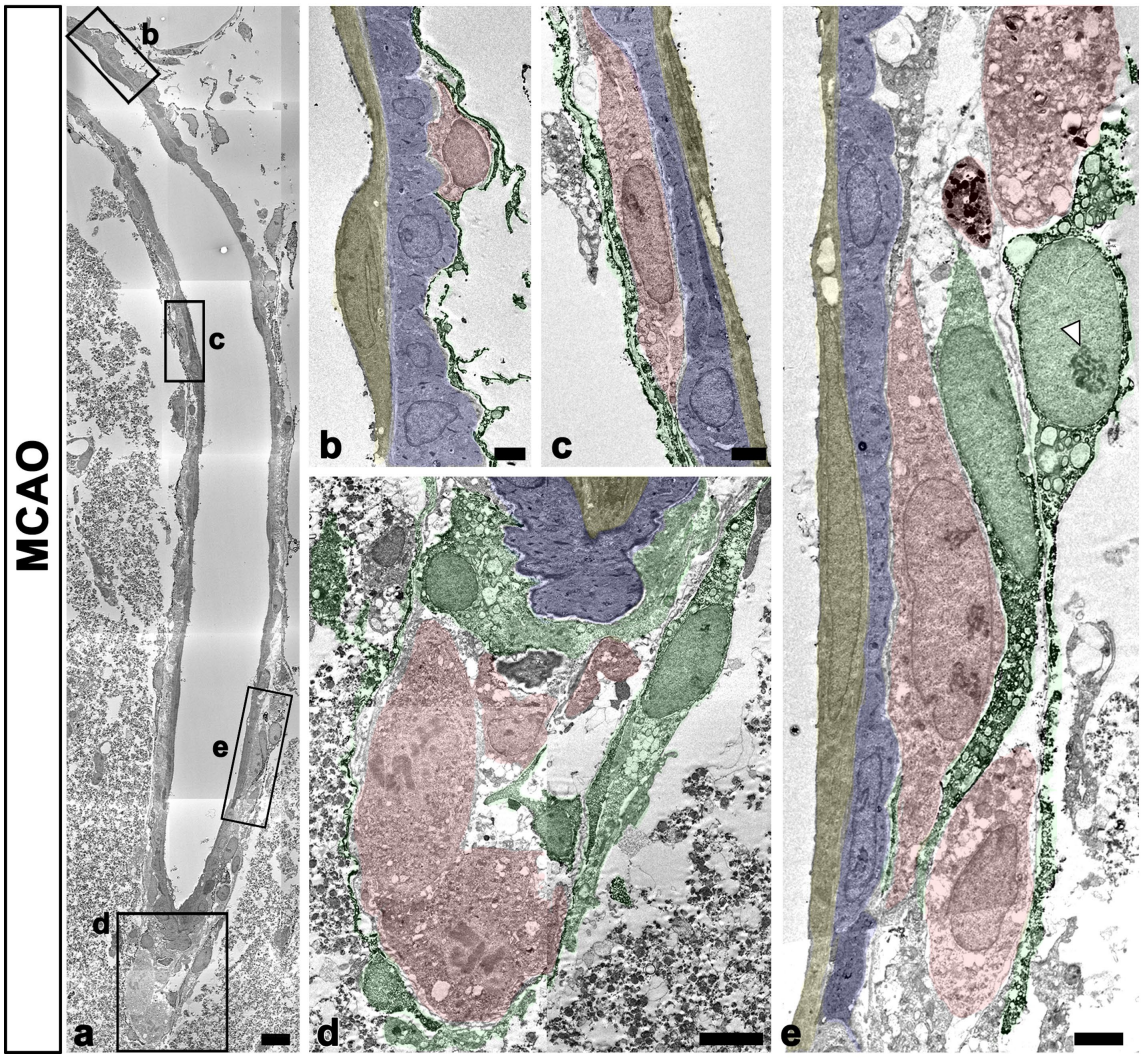
Ultrastructural characteristics of macrophages and PDGFR-β(+) cells within penetrating cortical vessels. **(A)** Lower magnification view showing the arteriole penetrating the ischemic cortex. The boxed areas in a are enlarged in **(B–E)**. Meningeal macrophages (shaded in red) are surrounded by a multilayered sheath of PDGFR-β-positive cells (shaded in green) that have euchromatic nuclei with prominent nucleoli (arrowhead in **E**) and dilated cisternae of the rough endoplasmic reticulum. Note that macrophages are located on the abluminal side of smooth muscle cells (shaded in blue) and endothelial cells (shaded in yellow) of penetrating arteriole. Scale bar = 10 μm for **(A)**; 2 μm for **(B,C,E)** and 5 μm for **(D)**.

**Figure 8:**
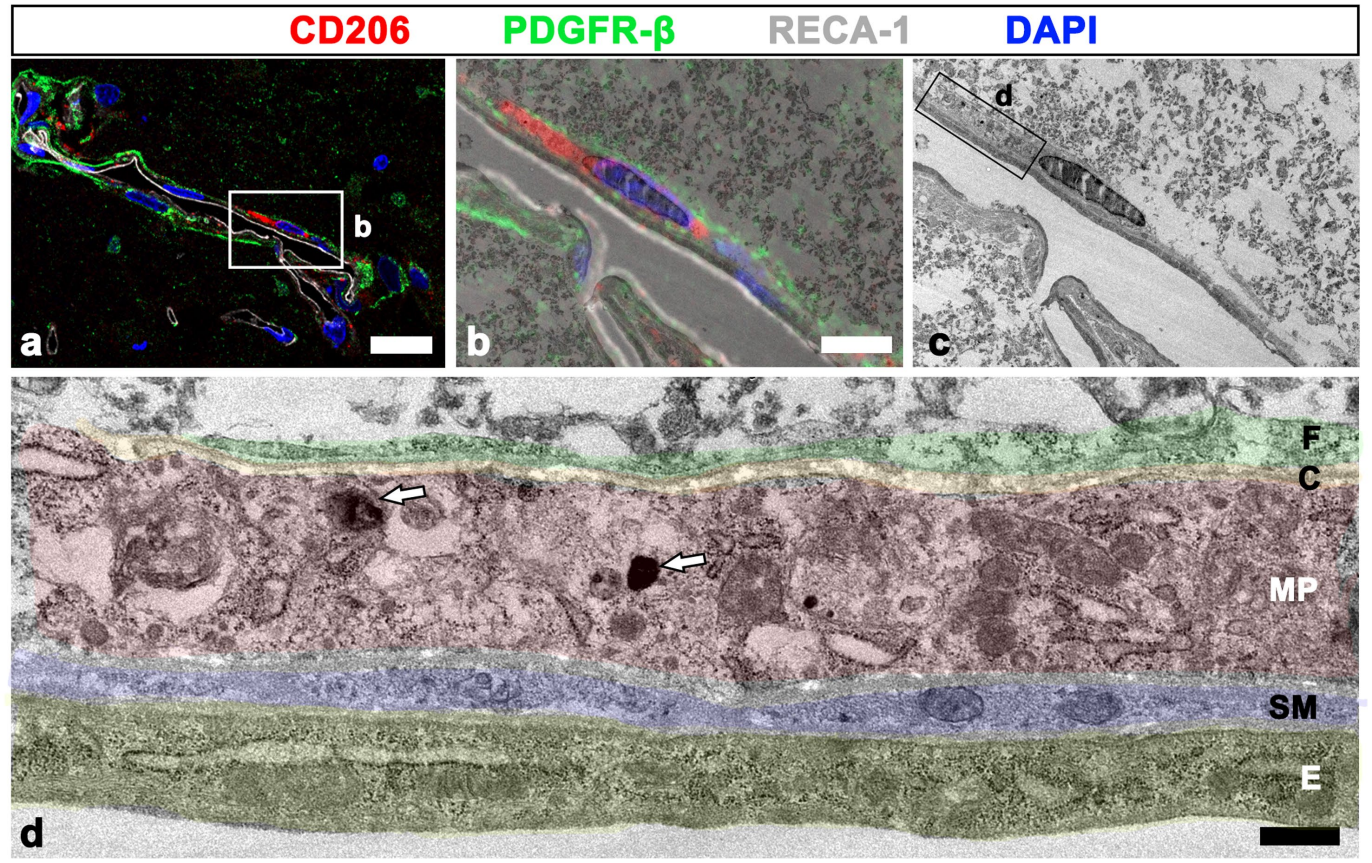
Relationship between meningeal macrophages and PDGFR-β-positive cells within penetrating vessels. **(A–C)** Confocal microscopic image of a semi-thin section triple labeled with CD206, PDGFR-β, and RECA-1 **(A)**, of a boxed area overlaid onto the corresponding electron microscopic image **(B)**, and the corresponding transmission electron microscopic image obtained from the same field **(C)**. **(D)** Higher magnification view of the boxed area in d showing that meningeal macrophages (MP; shaded in red) containing secondary lysosomes (white arrows in **D**) are localized outside the endothelial cells (**E**; shaded in orange) and smooth muscle cells (SM; shaded in blue) and are surrounded by PDGFR-β-positive cell processes (**F**; shaded in green) and collagen fibrils (**C**; shaded in yellow). Scale bar =20 μm for **(A)**; 5 μm for **(B–C)**; 0.5 μm for **(D)**.

The detailed ultrastructural characteristics of meningeal macrophages and PDGFR-β-positive cells within penetrating vessels in the infarcted cortex were further examined. Meningeal macrophages were invariably localized between smooth muscle cells and PDGFR-β-positive cells that had euchromatic nuclei with prominent nucleoli and dilated cisternae of rough endoplasmic reticulum, indicating active collagen synthesis (Figures 7A–E). In some parts of the arterial wall, PDGFR-β-positive cell bodies and processes formed a multilayered sheath, between which meningeal macrophages had close apposition with PDGFR-β-positive cells (Figure 7E). To more precisely determine the relationship between meningeal macrophages and PDGFR-β-positive cells within penetrating vessels, a correlative light and electron microscopy was performed. Semi-thin sections triple labeled with CD206, PDGFR-β, and RECA-1 were first observed using confocal microscopy, and the results clearly revealed that CD206-positive macrophages were localized between endothelial cells and PDGFR-β-positive cells (Figure 8A). Overlay of the confocal microscopy and transmission electron microscopy data confirmed that meningeal macrophage containing secondary lysosomes was localized outside the endothelial and smooth muscle cells and was surrounded by PDGFR-β-positive cell processes and collagen fibrils (Figures 8B–D).

**Figure 9:**
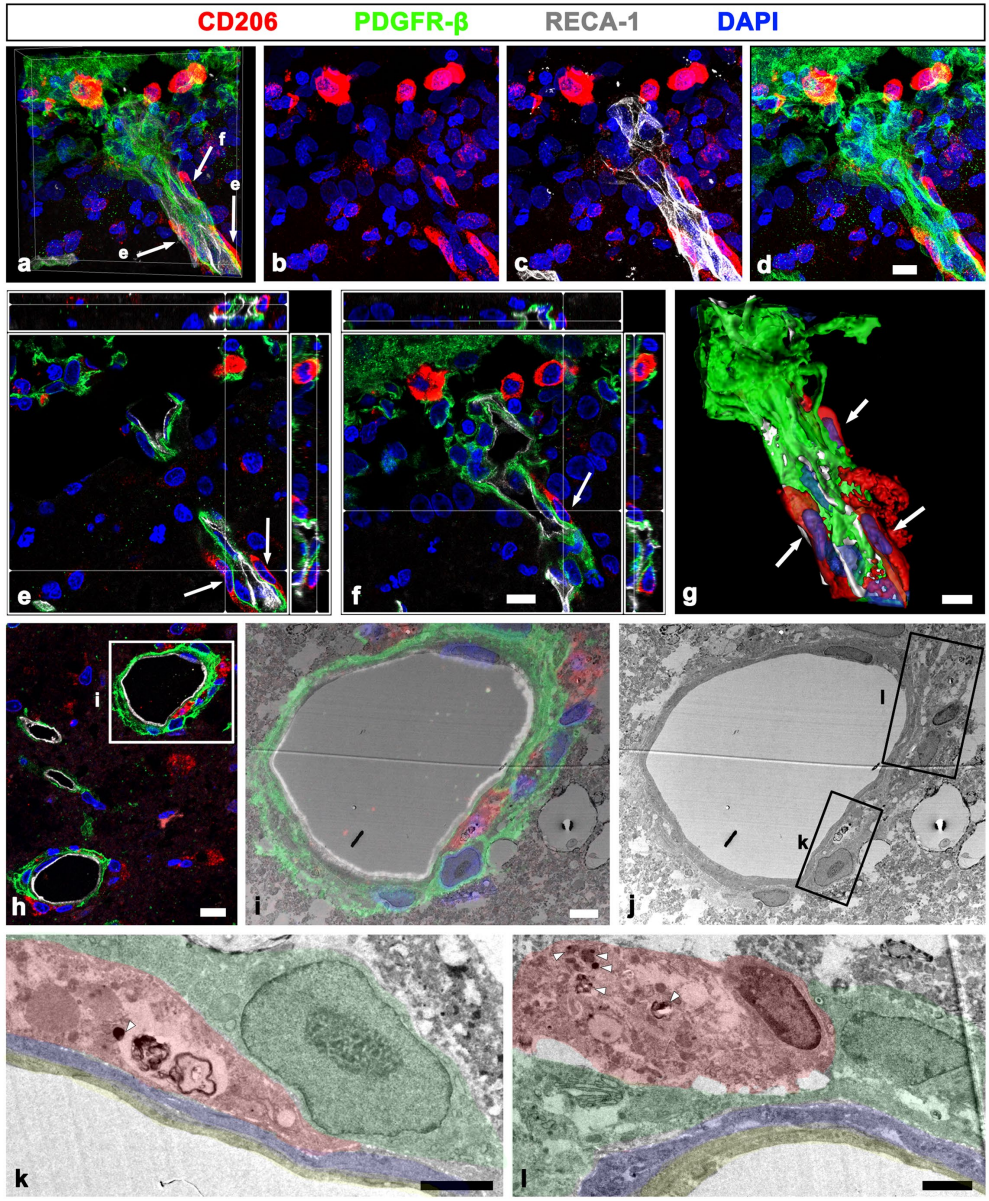
Localization of meningeal macrophages on both the adluminal and abluminal sides of PDGFR-β-positive cells related to penetrating vessels. **(A–G)** Triple labeling for PDGFR-β, CD206, and RECA-1 in MCAO rats at day 3 after reperfusion. **(A–D)** Several CD206-labeled macrophages are observed in the leptomeninges and within the cortical penetrating vessels. **(E,F)** Orthogonal views of three meningeal macrophages indicated by arrows in a showing that they are closely apposed to the outer part of PDGFR-β-positive cells within cortical penetrating vessels. **(G)** Three-dimensional magnified images showing that these meningeal macrophages (arrows) associated with penetrating vessels are localized on the abluminal side of PDGFR-β-positive cells. **(H–J)** Confocal microscopic image of a semi-thin section triple labeled with CD206, PDGFR-β, and RECA-1 **(H)**; of a boxed area overlaid onto the corresponding electron microscopic image **(I)**; and the corresponding transmission electron microscopic image obtained from the same field **(J)**. **(K,L)** Higher magnification views of the boxed areas in **(J)** showing that meningeal macrophages (shaded in red) containing secondary lysosomes (arrowheads in **K** and **L**) are localized not only between smooth muscle cells (shaded in blue) and PDGFR-β-positive cells (shaded in green), but also in close contact with the abluminal surface of PDGFR-β-positive cells. Endothelial cells are shaded in orange. Scale bar = 10 μm for **(A–F)**, 5 μm for **(G)**, 4 μm for **(I–J)**, and 2 μm for **(K–L)**.

In addition to their distribution in the perivascular space, CD206-positive macrophages were frequently observed on the abluminal side of PDGFR-β-positive perivascular fibroblasts, both closely adjacent or in direct contact (Figures 9A–D). Orthogonal magnified views (Figures 9E, F) and the three-dimensional reconstruction image (Figure 9G) clearly revealed that these macrophages were closely apposed to the outer part of PDGFR-β-positive fibroblasts. This finding was further supported by a correlative light and electron microscopy. Semi-thin sections triple labeled with CD 206, PDGFR-β, and RECA-1 revealed that CD206-positive macrophages were localized in both the adluminal and abluminal sides of PDGFR-β-positive cells associated with cortical vessels (Figure 9H). Overlay of the confocal microscopy and transmission electron microscopy data demonstrated that macrophages containing secondary lysosomes were located in close proximity to the abluminal surface of PDGFR-β-positive cells, as well as between the smooth muscle cells and PDGFR-β-positive cells (Figures 9I–L).

## 4. Discussion

This study provides the possible entry route of meningeal macrophages into the ischemic brain using a detailed spatial analysis of CD206+ macrophage infiltration in a rat model of stroke. First, the results showed that the number of CD206+ macrophages was significantly increased in both the leptomeninges and the underlying cerebral cortex, particularly along the wall of penetrating cortical vessels, 3 days after reperfusion. Next, inactive PDGFR-β(+) adventitial fibroblasts within the penetrating cortical vessels in sham-operated controls were activated upon stroke injury. The activation of these fibroblasts appeared to temporally overlap with the migration of meningeal macrophages along the penetrating cortical vessels in the lesion core. Finally, electron microscopic investigations showed that meningeal macrophages were localized in the perivascular Virchow–Robin space surrounding the vessels penetrating the cortical parenchyma. Particularly, macrophages were frequently located at the pia-lined extensions of subarachnoid space around the penetrating arterioles and ascending venules (i.e., the pial funnels), which were precisely identified by cells expressing PDGFR-β, a novel marker for leptomeningeal cells (Brinker et al., 2014; Gao et al., 2015). Within penetrating cortical vessels, macrophages were localized in the adluminal side of PDGFR-β(+) perivascular fibroblasts, with these cells being closely associated with each other. In addition, macrophages were frequently observed in the abluminal surface of these PDGFR-β(+) fibroblasts. These novel observations suggest that the perivascular Virchow–Robin space of penetrating vessels is an entry route of meningeal macrophages from the subarachnoid space into the infarcted brain parenchyma and that activation of PDGFR-β-positive adventitial fibroblasts is involved in their infiltration. Furthermore, the present study suggests that PDGFR-β can be potentially used as a novel marker to clearly delineate the Virchow–Robin space, supporting our previous work regarding the detailed ultrastructural evidence of PDGFR-β as an appropriate marker of leptomeningeal cells and perivascular adventitial cells (Riew et al., 2018, 2020).

We recently found that PDGFR-β-positive leptomeningeal fibroblasts ensheathing the leptomeningeal vessels abruptly converted into inactive fibrocytes ensheathing the penetrating cerebral vasculature, despite both being regarded as adventitial fibroblasts (Riew et al., 2020). Notably, inactive PDGFR-β-positive adventitial cells within the penetrating cortical vessels in sham-operated controls were activated upon stroke injury, altering their morphology and adapting molecular features of leptomeningeal PDGFR-β-positive fibroblasts. The expressions of the intermediate filaments nestin and vimentin and of type I and type IV collagens were significantly increased in these vascular PDGFR-β(+) cells. Along with the ultrastructural characteristics of euchromatic nuclei, prominent rough endoplasmic reticulum, and association with collagen fibrils, these molecular signatures indicate that the fibroblasts undergo active collagen synthesis, as described previously (Riew et al., 2018, 2020). Interestingly, activation of PDGFR-β(+) adventitial cells of penetrating vessels correlated temporally and spatially with infiltration of meningeal macrophages into their perivascular space in the infarcted cortex. Several studies including ours have shown a close relationship between macrophages and fibroblasts in the fibrotic scar formation following CNS injury, including in MCAO (Kelly et al., 2016); spinal cord injury (Zhu et al., 2015); and acute striatal injury (Riew et al., 2018). Shibahara et al. recently showed that after permanent MCAO, PDGFR-β-positive cells expressed C-C motif ligand 2 (a chemokine recruiter) and colony-stimulating factor 1 (a proliferating factor of monocytes/macrophages). This suggested that the infiltration of macrophages into the infarct area is actively induced by PDGFRβ-positive pericytes covering endothelial cells within the infarct area (Shibahara et al., 2020). Thus, close cell-to-cell contacts between meningeal macrophages and PDGFR-β (+) adventitial cells within penetrating cortical vessels may be essential for recruiting the meningeal macrophages from the subarachnoid space into the perivascular space. However, further studies are needed to elucidate the molecular mechanisms underlying the recruitment of meningeal macrophages.

Recent studies support the importance of meningeal immunity in neuroinflammation and neurodegenerative diseases in addition to its structural support and protection for the CNS (Rua and McGavern, 2018; Alves de Lima et al., 2020; Derk et al., 2021). Meningeal macrophages, a major population of leptomeningeal immune cells, belong to BAMs. BAMs are yolk-sac-derived non-parenchymal brain macrophages localized in the leptomeninges, perivascular space, and choroid plexus (Goldmann et al., 2016; Mrdjen et al., 2018; Van Hove et al., 2019). Under physiological conditions, BAMs contribute to vascular barrier function, perivascular filtering, drainage system, antigen recognition and presentation, and phagocytosis (Kierdorf et al., 2019; Yang et al., 2019; Prinz et al., 2021). Accumulating evidence supports the active roles of BAMs in various brain pathologies owing to their anatomical characteristics: aligned between vascular walls and located at the interface between the brain parenchyma and peripheral immune system (Hawkes and McLaurin, 2009; Schläger et al., 2016; Faraco et al., 2017; Park et al., 2017; Mrdjen et al., 2018; Kierdorf et al., 2019; Yang et al., 2019; Rajan et al., 2020; Wan et al., 2021; Gerganova et al., 2022). Particularly, Pedragosa et al. reported that BAMs participate in increasing vascular permeability, facilitating granulocyte recruitment, and contributing to neurological dysfunction in the acute phase of ischemic stroke (Pedragosa et al., 2018).

A recent experiment using CD163 as a marker for rat BAMs revealed that the number of BAMs was increased within meninges, perivascular spaces, and ischemic parenchyma 3 days after stroke (Rajan et al., 2020). Consistent with these findings, our data show that CD206/Iba1 double-labeled macrophages were not detectable in cortical parenchyma of sham-operated rats, but they were increased in the leptomeninges and within the penetrating large cortical vessels after MCAO. The increase of these macrophages in these areas appeared to precede their accumulation in the infarcted cortex. Macrophages were confined to the superficial cortical parenchyma in close proximity to the pia mater and were often located in the abluminal sides of PDGFR-β(+) perivascular fibroblasts associated with penetrating cortical vessels. These findings suggested that macrophages in the infarcted cortex originated from the meningeal macrophages *via* the Virchow–Robin space. However, we cannot exclude the possibility that the breakdown of the glia limitans superficialis after MCAO may lead to the recruitment of meningeal macrophages from the leptomeninges. However, bone marrow-derived macrophage acquired a BAM phenotype in an experimental autoimmune encephalomyelitis (EAE) model (Jordão et al., 2019). This was further supported by a bone marrow transplantation study using chimeric mice after ischemic stroke (Rajan et al., 2020). As such, some of the CD206-positive macrophages accumulating in the ischemic cortex might originate from the parenchymal microglia or bone marrow-derived monocytes. However, unlike our study, these studies were based on CD163-immunosorted cells or the whole brain; thus, the detailed anatomical localization and different marker gene expression (i.e., CD206, Lyve1, major histocompatibility complex class II) of these BAM subsets and other myeloid cells need to be clarified. In addition, further investigations are needed to determine whether BAMs continue to accumulate in the subacute to chronic phase of stroke and to determine their roles in tissue remodeling processes.

Diverse immune cells at the CNS borders and brain-resident parenchymal microglia infiltrate the lesion core in response to ischemic insults (Iadecola et al., 2020). In addition to transendothelial migration of leukocytes into the ischemic brain throughout the compromised blood–brain barrier, their alternative cerebral infiltration route from the leptomeninges *via* the Virchow–Robin space has also been reported. In both rodents and humans, neutrophils were identified in the leptomeninges and Virchow–Robin space as early as a few hours after stroke (Perez-de-Puig et al., 2015). This was further corroborated by a study in which injected fluorescent neutrophils extravasated from subpial vessels to reach the ischemic parenchyma in MCAO mice (Otxoa-de-Amezaga et al., 2019). In addition, T-cell infiltration into the leptomeninges *via* the pial vasculature was observed in an EAE mouse model (Schläger et al., 2016). Further, immune surveillance of brain perivascular spaces by macrophages was reported previously (Bechmann et al., 2001). Collectively, these findings suggest the Virchow–Robin space as a route for infiltration of diverse immune cells into the brain parenchyma during CNS injury. However, to the best of our knowledge, this study is the first to ultrastructurally characterize this route for meningeal macrophages into the ischemic cortex.

## 5. Conclusion

**Figure 10:**
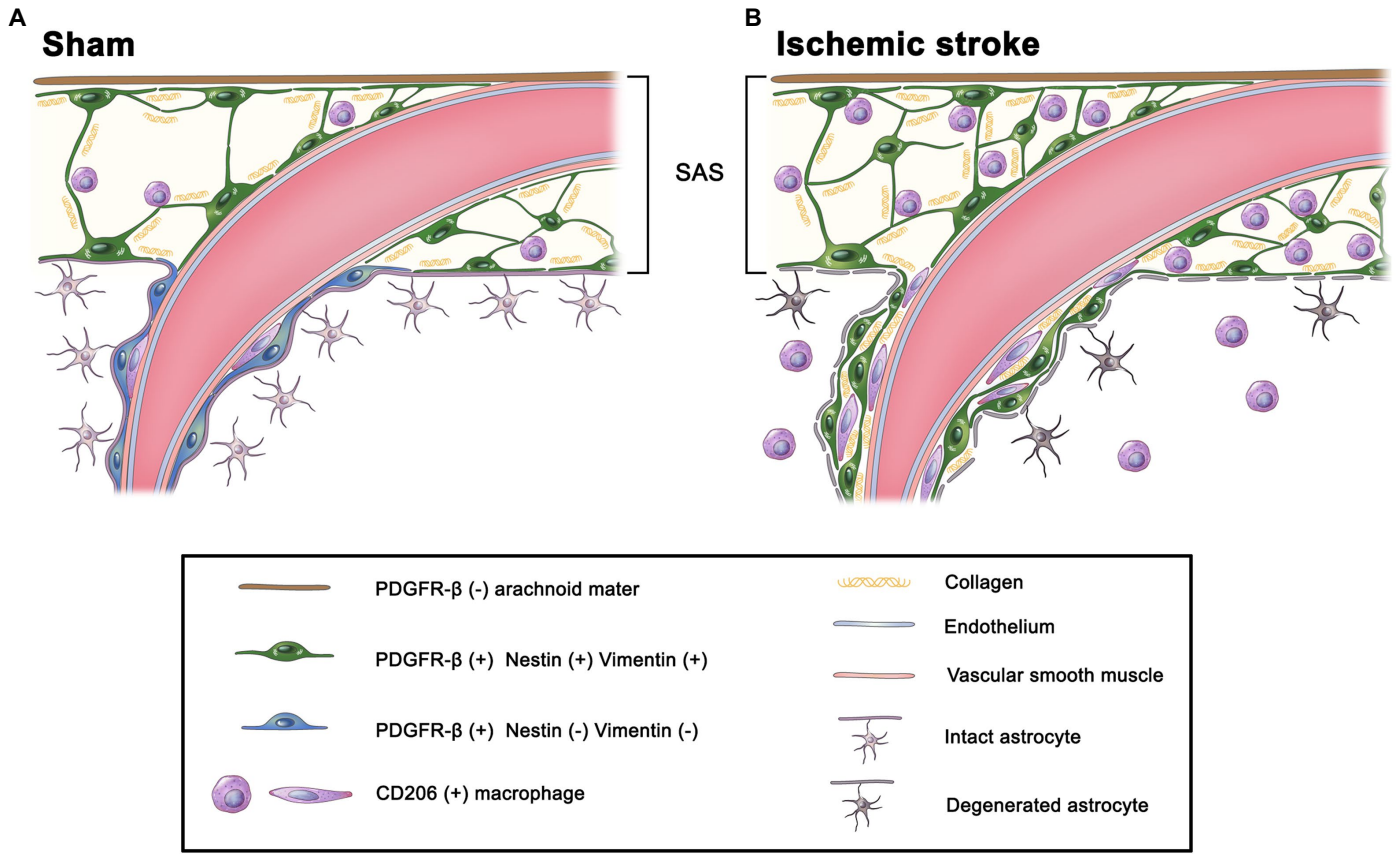
Schematic diagram of the potential infiltration route for meningeal macrophages into the infarct cortex. **(A)** In the sham-operated rats, meningeal macrophages are mostly localized within the leptomeninges and are occasionally located within the perivascular space of large cortical penetrating vessels, but they are not detected in the cortical parenchyma. Although PDGFR-β-positive leptomeningeal cells ensheathing leptomeningeal vessels express the intermediate filaments nestin and vimentin and are closely apposed to collagen fibrils, PDGFR-β-positive perivascular cells within penetrating cortical vessels are more flattened with loss of nestin and vimentin. **(B)** At 3 days after MCAO, PDGFR-β(+) perivascular adventitial cells within the penetrating cortical vessels are activated upon stroke injury, altering their morphology, adapting molecular features of leptomeningeal PDGFR-β(+) fibroblasts by expressing nestin and vimentin, and being apposed to collagen fibrils. In addition, meningeal macrophages are commonly found along the perivascular space (i.e., Virchow–Robin space) of the penetrating cortical vessels, where they are closely associated with activated PDGFR-β(+) cells. This indicates that the Virchow–Robin space is an entry route through which macrophages migrate from the leptomeninges into the infarcted cortex.

In a rat model of MCAO-induced brain ischemia, PDGFR-β-positive perivascular adventitial fibroblasts within the penetrating cortical vessels have morphological and molecular characteristics similar to those of leptomeningeal cells with phenotypes of active fibroblasts. The activation of these PDGFR-β-positive cells temporally and spatially correlated with the infiltration of meningeal macrophages into the Virchow–Robin space surrounding the penetrating cortical vessels, with both cells showing close spatial association with each other (Figure 10). Collectively, our data provide novel morphological evidence that the perivascular Virchow–Robin space surrounding the penetrating cortical vessels could be an entry route of meningeal macrophages from the subarachnoid space into the ischemic cortical parenchyma. Further, activated PDGFR-β-positive adventitial fibroblasts are involved in this process.

## Data availability statement

The raw data supporting the conclusions of this article will be made available by the authors, without undue reservation.

## Ethics statement

The animal study was reviewed and approved by the IACUC (Institutional Animal Care and Use Committee) at the College of Medicine of The Catholic University of Korea (Approval number: CUMS-2020-0041-01).

## Author contributions

T-RR contributed to the animal modeling, immunohistochemistry, immunoelectron microscopy, and quantitative and qualitative image analysis. J-WH and XJ contributed to the animal modeling and immunohistochemistry. HK worked on the electron microscopy. M-YL worked on the design of the study, data analysis, and final manuscript preparation. All authors have contributed significantly to the research and the article preparation. All authors contributed to the article and approved the submitted version.

## Funding

This research was supported by the grant from the National Research Foundation of Korea (NRF; grant number NRF-2020R1A2B5B01001442).

## Conflict of interest

The authors declare that the research was conducted in the absence of any commercial or financial relationships that could be construed as a potential conflict of interest.

## Publisher’s note

All claims expressed in this article are solely those of the authors and do not necessarily represent those of their affiliated organizations, or those of the publisher, the editors and the reviewers. Any product that may be evaluated in this article, or claim that may be made by its manufacturer, is not guaranteed or endorsed by the publisher.

## Supplementary material

SUPPLEMENTARY FIGURE 1 Validation of two markers for CNS border-associated macrophages: CD206 and lymphatic vessel endothelial hyaluronan receptor 1 (Lyve1). (a–f) Double labeling with either CD206 or Lyve1 and Iba1 in control sections showing that CD206 and Iba1 generally overlap within leptomeninges and cortical vessels, while most Iba1-positive cells do not express Lyve1 (arrows in d and e). Note that both markers are not detected in Iba1-positive cells with clearly demarcated processes in the cortical parenchyma. (g–i) Double-labeling with CD206 and Lyve1 in control sections showing that Lyve1 expression is restricted to a subset of CD206-positive macrophages within leptomeninges. Dashed lines in c, f, and i represent the boundary separating the leptomeninges from the cortical parenchyma. (j, k) Quantitative analysis showing that the number of CD206/Iba1 double-labeled macrophages within leptomeninges and cortical vessels is markedly higher than that of Lyve1/Iba1 double-labeled macrophages (n=7 or 10 rats, Student’s t-test). Data are expressed as the mean ± SEM, and numbers on each bar graph indicate p values. Scale bar = 50 μm for a–i.

SUPPLEMENTARY FIGURE 2 CD206-positive macrophages in the underlying cerebral cortex of sham-operated and MCAO rats. (a–d) Higher magnification views of the boxed area in Figure 1c. Triple labeling for CD206, the endothelial cell marker RECA-1, and Iba1 in control sections showing that CD206/Iba1 double-labeled macrophages are not observed in the cortical parenchyma. (e–h) Higher magnification views of the boxed area in Figure 1f. Triple labeling for CD206, RECA-1, and Iba1 in the cortex reperfused for 3 days showing that CD206/Iba1 double-labeled macrophages have accumulated in the superficial cortical parenchyma in close proximity to the pia mater. Dashed lines in a and e represent the boundary separating the leptomeninges from the cortical parenchyma. Scale bar = 10 μm for a–h.

SUPPLEMENTARY FIGURE 3 Molecular characterization of PDGFR-β expression in the leptomeninges and underlying cortex of sham-operated and MCAO rats. (a–f) Double labeling for PDGFR-β and type I collagen in sham-operated (a–c) and MCAO rats at day 3 after reperfusion (d–f). Note that PDGFR-β and collagen I share overlapping spatial profiles within the leptomeninges and cortical blood vessels in both sham-operated and MCAO rats. Dashed lines in c and f represent the boundary separating the leptomeninges from the cortical parenchyma. Scale bar = 50 μm for a–f.

SUPPLEMENTARY FIGURE 4 Molecular coincidence between CD206-positive macrophages and PDGFR-β expression in cortical penetrating vessels of sham-operated and MCAO rats. (a–h) Double labeling for PDGFR-β and CD206 showing that CD206-positive macrophages are distributed only in a small fraction of the vasculature occupied by PDGFR-β in both sham-operated (a–d) and MCAO rats at day 3 after reperfusion (e–h). (i) Quantitative analysis showing that the vascular length occupied by CD206 is significantly higher than that of PDGFR-β-positive vessels within the cortical parenchyma in both sham-operated and MCAO rats (n = 6–9 rats; one-way ANOVA with Tukey’s multiple comparison test). Data are expressed as the mean ± SEM, and numbers on each bar graph indicate p values. Scale bar = 50 μm for a–h.

## Abbreviations

CNS: Central nervous system
Iba1: Lonized calcium-binding adaptor molecule 1
Lyve1: Lymphatic vessel endothelial hyaluronan receptor 1
MCAO: Middle cerebral artery occlusion
PB: Phosphate buffer
PDGFR-β: Platelet-derived growth factor beta receptor
RECA-1: Rat endothelial cell antigen-1
