# Supplementary figures and images for: Infiltration of meningeal macrophages into the Virchow–Robin space after ischemic stroke in rats: Correlation with activated PDGFR-β-positive adventitial fibroblasts
Source: Front Mol Neurosci. 2022 Dec 30;15:1033271. doi: 10.3389/fnmol.2022.1033271 (PMC9837109; doi:10.3389/fnmol.2022.1033271)

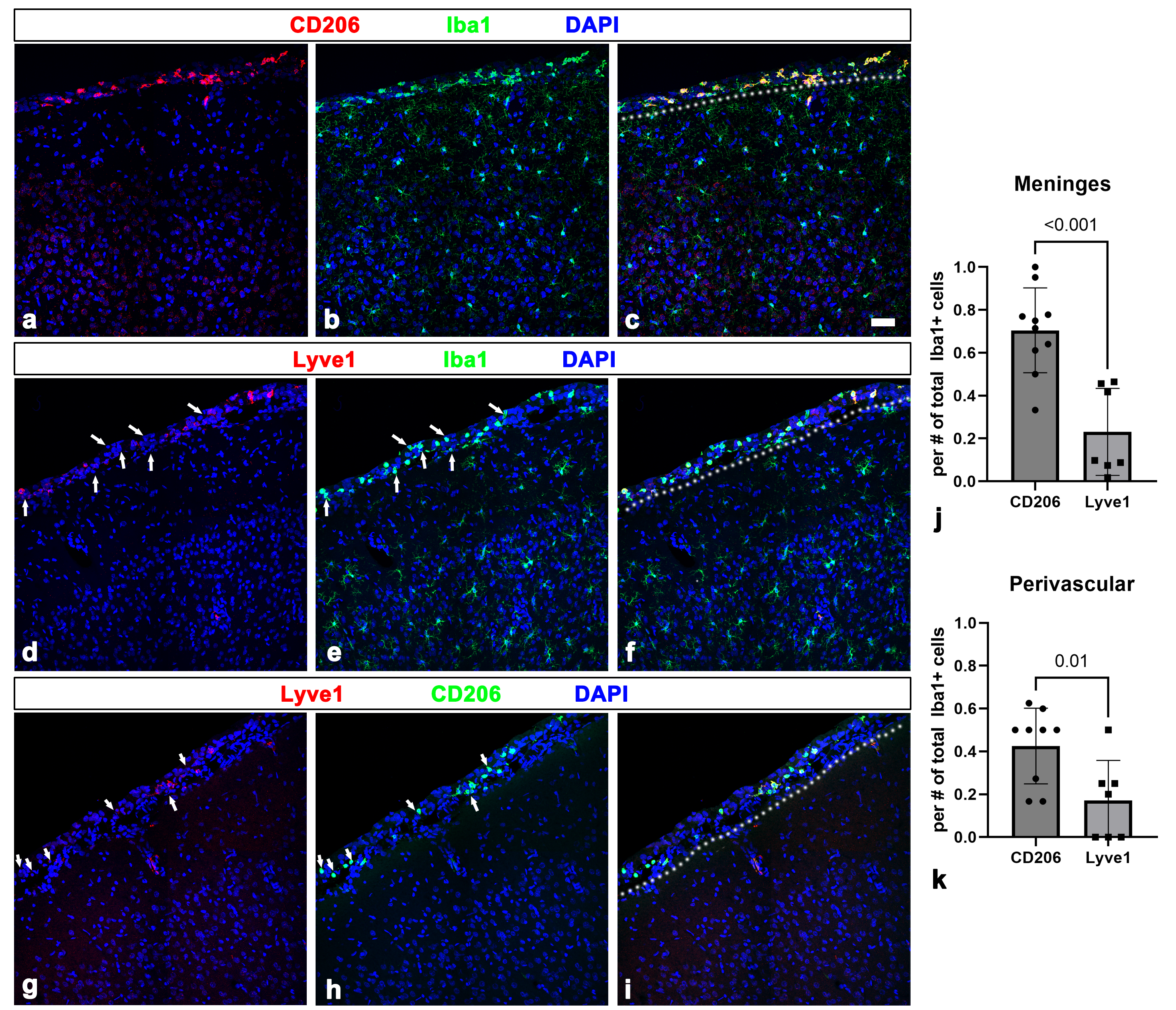

Supplement: SUPPLEMENTARY FIGURE 1 — Validation of two markers for CNS border-associated macrophages: CD206 and lymphatic vessel endothelial hyaluronan receptor 1 (Lyve1). (a–f) Double labeling with either CD206 or Lyve1 and Iba1 in control sections showing that CD206 and Iba1 generally overlap within leptomeninges and cortical vessels, while most Iba1-positive cells do not express Lyve1 (arrows in d and e). Note that both markers are not detected in Iba1-positive cells with clearly demarcated processes in the cortical parenchyma. (g–i) Double-labeling with CD206 and Lyve1 in control sections showing that Lyve1 expression is restricted to a subset of CD206-positive macrophages within leptomeninges. Dashed lines in c, f, and i represent the boundary separating the leptomeninges from the cortical parenchyma. (j, k) Quantitative analysis showing that the number of CD206/Iba1 double-labeled macrophages within leptomeninges and cortical vessels is markedly higher than that of Lyve1/Iba1 double-labeled macrophages (n=7 or 10 rats, Student’s t-test). Data are expressed as the mean ± SEM, and numbers on each bar graph indicate p values. Scale bar = 50 μm for a–i. [file Image_1.TIF]

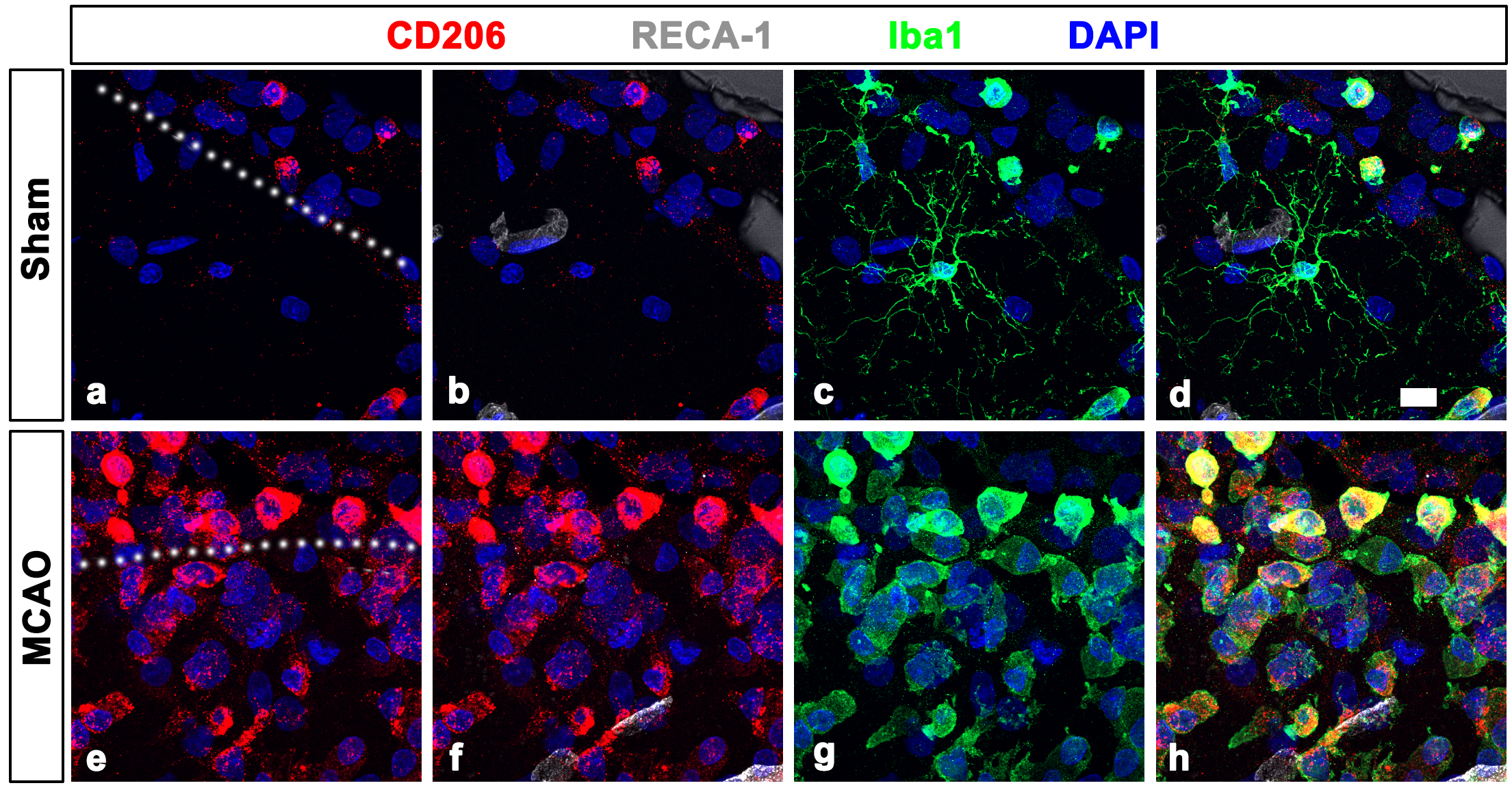

Supplement: SUPPLEMENTARY FIGURE 2 — CD206-positive macrophages in the underlying cerebral cortex of sham-operated and MCAO rats. (a–d) Higher magnification views of the boxed area in Figure 1c. Triple labeling for CD206, the endothelial cell marker RECA-1, and Iba1 in control sections showing that CD206/Iba1 double-labeled macrophages are not observed in the cortical parenchyma. (e–h) Higher magnification views of the boxed area in Figure 1f. Triple labeling for CD206, RECA-1, and Iba1 in the cortex reperfused for 3 days showing that CD206/Iba1 double-labeled macrophages have accumulated in the superficial cortical parenchyma in close proximity to the pia mater. Dashed lines in a and e represent the boundary separating the leptomeninges from the cortical parenchyma. Scale bar = 10 μm for a–h. [file Image_2.TIF]

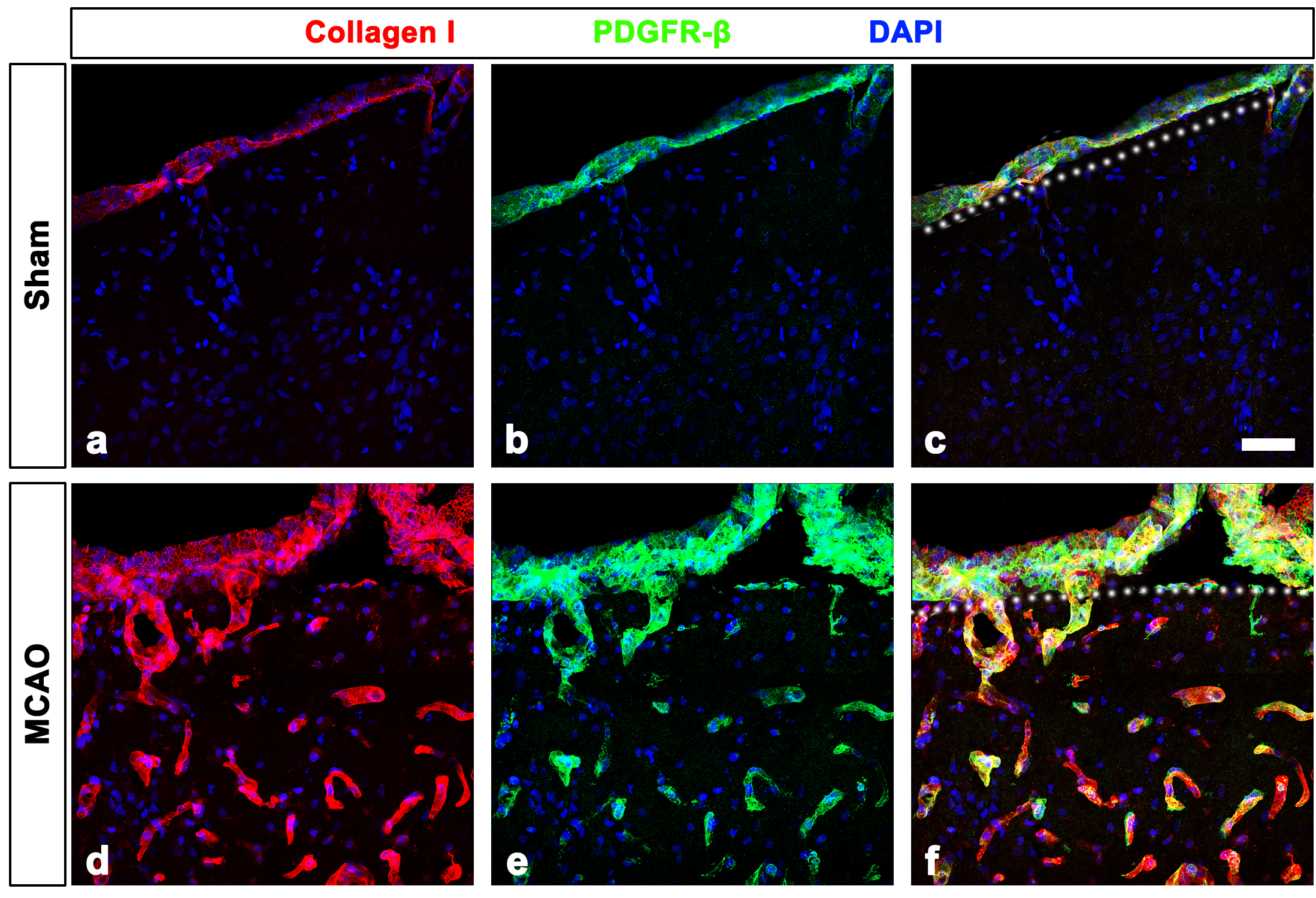

Supplement: SUPPLEMENTARY FIGURE 3 — Molecular characterization of PDGFR-β expression in the leptomeninges and underlying cortex of sham-operated and MCAO rats. (a–f) Double labeling for PDGFR-β and type I collagen in sham-operated (a–c) and MCAO rats at day 3 after reperfusion (d–f). Note that PDGFR-β and collagen I share overlapping spatial profiles within the leptomeninges and cortical blood vessels in both sham-operated and MCAO rats. Dashed lines in c and f represent the boundary separating the leptomeninges from the cortical parenchyma. Scale bar = 50 μm for a–f. [file Image_3.TIF]

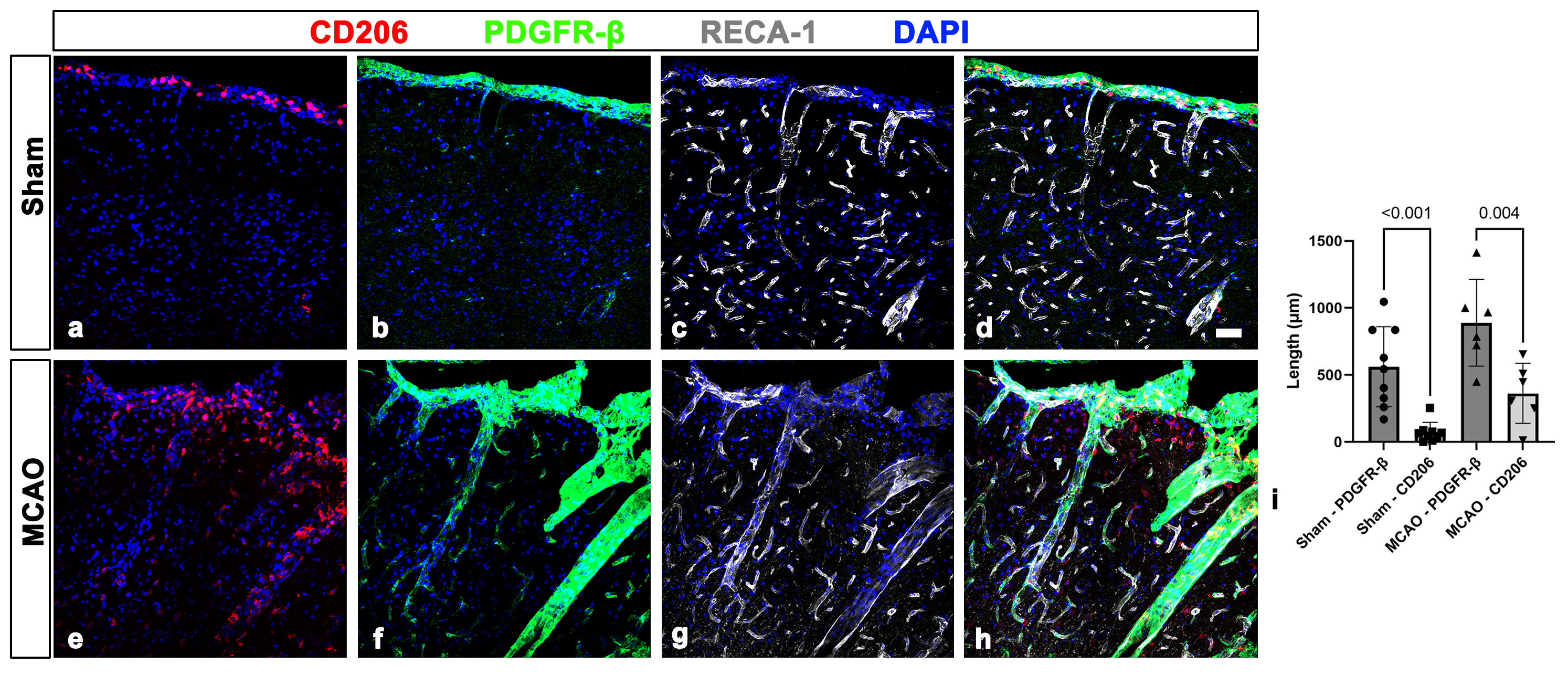

Supplement: SUPPLEMENTARY FIGURE 4 — Molecular coincidence between CD206-positive macrophages and PDGFR-β expression in cortical penetrating vessels of sham-operated and MCAO rats. (a–h) Double labeling for PDGFR-β and CD206 showing that CD206-positive macrophages are distributed only in a small fraction of the vasculature occupied by PDGFR-β in both sham-operated (a–d) and MCAO rats at day 3 after reperfusion (e–h). (i) Quantitative analysis showing that the vascular length occupied by CD206 is significantly higher than that of PDGFR-β-positive vessels within the cortical parenchyma in both sham-operated and MCAO rats (n = 6–9 rats; one-way ANOVA with Tukey’s multiple comparison test). Data are expressed as the mean ± SEM, and numbers on each bar graph indicate p values. Scale bar = 50 μm for a–h. [file Image_4.TIF]
